# A gel-free approach in vascular smooth muscle cell proteome: perspectives for a better insight into activation

**DOI:** 10.1186/1477-5956-8-15

**Published:** 2010-03-24

**Authors:** Silvia Rocchiccioli, Lorenzo Citti, Claudia Boccardi, Nadia Ucciferri, Lorena Tedeschi, Caterina Lande, Maria Giovanna Trivella, Antonella Cecchettini

**Affiliations:** 1 Institute of Clinical Physiology, CNR, Via Moruzzi 1, 56124 Pisa, Italy; 2 Department of Human Morphology and Applied Biology, University of Pisa, Via Roma 55, 56126 Pisa, Italy

## Abstract

**Background:**

The use of chromatography coupled with mass spectrometry (MS) analysis is a powerful approach to identify proteins, owing to its capacity to fractionate molecules according to different chemical features. The first protein expression map of vascular smooth muscle cells (VSMC) was published in 2001 and since then other papers have been produced. The most detailed two-dimensional polyacrylamide gel electrophoresis (2D-PAGE) map was presented by Mayr et al who identified 235 proteins, corresponding to the 154 most abundant unique proteins in mouse aortic VSMC. A chromatographic approach aimed at fractionating the VSMC proteome has never been used before.

**Results:**

This paper describes a strategy for the study of the VSMC proteome. Our approach was based on pre-fractionation with ion exchange chromatography coupled with matrix assisted laser desorption-time of flight mass spectrometry analysis assisted by a liquid chromatography (LC-MALDI-TOF/TOF). Ion exchange chromatography resulted in a good strategy designed to simplify the complexity of the cellular extract and to identify a large number of proteins. Selectivity based on the ion-exchange chemical features was adequate if evaluated on the basis of protein pI. The LC-MALDI approach proved to be highly reproducible and sensitive since we were able to identify up to 815 proteins with a concentration dynamic range of 7 orders of magnitude.

**Conclusions:**

In our opinion, the large number of identified proteins and the promising quantitative reproducibility made this approach a powerful method to analyze complex protein mixtures in a high throughput way and to obtain statistical data for the discovery of key factors involved in VSMC activation and to analyze a label-free differential protein expression.

## Background

The use of chromatography coupled with MS analysis is a powerful approach for the identification of proteins, owing to its capacity to fractionate molecules with different chemical features [1-4]. Furthermore, LC-MALDI-TOF/TOF analysis combined with preliminary fractionation of a total protein extract is a potential tool for biomarker discovery because of its high sensitivity and high throughput capacity [5].

However, the use of LC-MALDI analysis still needs to be optimized and evaluated [6,7]. To obtain useful information for comparative analysis of samples and differential protein expression using a label-free approach in LC-MALDI techniques, the reproducibility in measuring m/z abundances (peak intensity) and a linear relation between intensity and marker concentration are essential [8-10]. Moreover, although LC-MALDI MS/MS analysis is a high mass precision technique, it is time consuming, especially if applied to a large number of samples. For this reason a good compromise between sample pre-processing and high performance liquid chromatography (HPLC) separation would be necessary to avoid the masking effect of high abundant proteins and to evidence hypothetical biomarkers.

We set up and assessed a strategy for rapid data collection coupled with good reproducibility and precision. We checked this approach in the very interesting model system represented by vascular smooth muscle cells (VSMC). The cells are characterized by the capability of switching from a contractile and completely differentiated to a proliferating, migratory phenotype. This change is mainly due to the stimulation from growth factors and cytokines that are responsible for cell activation. Activated VSMCs play a pivotal role in the onset and progression of cardiovascular diseases, causing the development of atheromatous plaque and restenotic lesions [11]. Therefore, great attention has been focussed on the study of these cells, in order to find the factors involved in the activation process and/or the biomarkers of the activated, pathological phenotype, since these factors could be the putative targets of specific, innovative therapeutic strategies. In this respect, a proteomic approach is necessary but, up until now, only a few works have been realized and the great majority carried out with the use of 2D-PAGE. The first map of VSMC protein expression was published in 2001 [12] and since then other papers have been produced [13-17]. The most detailed 2D-PAGE map was presented by Mayr et al [18] who identified 235 proteins, corresponding to the 154 most abundant unique proteins, in mouse aortic VSMC. However, this technique is slow, hindered by the limited dynamic range and not sufficiently sensitive, especially for the study of hypothetical biomarkers, which are likely to be expressed in very low concentrations.

For all these reasons, we streamlined a non-conventional, sensitive and reproducible proteomic strategy that allowed us to identify 815 non-redundant VSMC proteins. This approach can be easily adapted for more detailed research and exploited for future analyses in biomarker discovery.

## Results and Discussion

**Figure 1:**
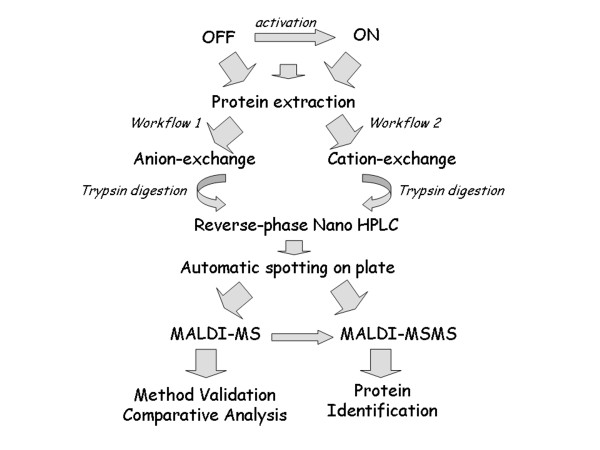
**Experimental procedure**. It summarizes the experimental procedure we adopted for our study. Proteins from ON- OFF- and 10'-VSMC were fractionated by ion-exchange chromatography (cation and anion), the 'specific sample solution' was tryptic digested and the peptides were separated by reverse-phase nano-HPLC before MALDI analysis.

VSMCs cultured under standard conditions with 10% FBS display a characteristic synthetic, activated phenotype (ON-VSMC) endowed with the ability of migrating and proliferating. Otherwise, when left in serum-free medium for three days or more, they assume a differentiated quiescent phenotype (OFF-VSMC) able to contract [19]. Serum growth factors and cytokines are known to be in charge of this phenotypic switch; moreover, this process is responsible for the onset and progression of the principal cardiovascular diseases. In order to optimize methods for the analysis of the activation mechanism which can eventually be exploited for biomarker discovery, we analyzed ON- and OFF-VSMC and also cells maintained for three days in absence of serum and then activated for 10 minutes with fetal bovine serum (FBS) (10'-VSMC). The effective change in cellular phenotype was checked as reported in the literature [20]. For all three protein extracts the workflow we adopted to analyze the VSMC proteome is reported in figure 1.

Aim of ion-exchange chromatography is to simplify the total proteome of the cell extract and to avoid the masking effect due to the excessive complexity and presence of highly abundant proteins [21].

**Figure 2:**
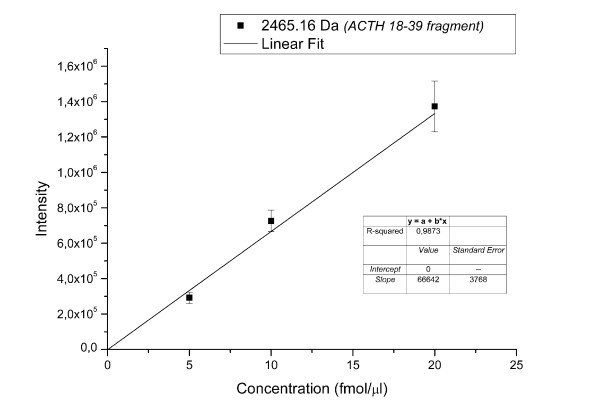
**Plot Concentration/Area signal of ACTH standard peptide**. It shows the plot of the area/concentration of a standard peptide (ACTH 18-39 clip, 2465.16 Da) after MALDI-MS analysis by 5000 shots/spot. We performed four replicated runs for each known concentration (5, 10 and 20 fmol/μl) of the standard peptide.

After sample pre-processing (anion- and cation-exchange resins), a relatively short HPLC run of a 30 min gradient was chosen in order to obtain a suitable separation of the peptides and to avoid peptide redundancy in contiguous spots due to an inefficient gradient rate. The fractions between 10 and 50 minutes were spotted resulting in approximately 200 spots per sample, but a preliminary analysis evidenced that only the first 150 fractions were informative. This approach allowed the spotting of up to 6 samples (all samples of ON, OFF, 10' for anion- and cation-exchange chromatography) on a single MALDI plate minimizing plate to plate signal variability, cost and time. The spots were analyzed using a 4800 MALDI-TOF/TOF mass spectrometer and the MS/MS data were pre-processed by the manufacturer computer program GPS Explorer and identified using the MASCOT search engine. To evaluate the possibility of comparing "control" and "activated" samples through the peptide area and to estimate the "quantitative" reproducibility of the MALDI signal intensity, we applied four replicated runs and we used an internal standard of known concentration (ACTH 18-39 fragment) diluted in the matrix (Figure 2). A significant correlation was found between spiked peptide concentration and intensity in the range of 5 to 20 fmol/μl. The precision of the measure evaluated as mean CV% between the four replicates was 9.95 ± 1.35%. As a matter of fact, even though MALDI ionization is known to yield poor reproducibility of peak intensity due to the heterogeneous co-crystallization of the matrix and the peptides [7], a high shot count (we used 5000 shots/spectra) could lead to greater reproducibility, as reported in the literature by Hattan et al [9].

To evaluate the efficiency of the ion-exchange resins we checked pI distribution of the identified proteins. We divided the identified proteins into three different pI ranges (3.69-8.99; 9.06-9.99 and 10-12.02) according to the acidic fraction, while for the cation-exchange the three pI fractions were 3.76-4.98; 5-5.98 and 6.01-12.02. Resin performance in terms of selectivity based on ionic properties proved to be very good, since 80% of the proteins bound to the anionic resin displayed an acid pI (pI < 8) and 60% of the proteins isolated by the cation-exchange showed basic pI (pI>6). Furthermore, 73% of these basic proteins had pI > 8, indicating that this approach could be used to complement 2D-gel analysis where high pI proteins are under-represented [22].

A total of 815 unique proteins, each of them present in all technical replicates, has been obtained and the complete list is reported in "Table-TotalUniqueProteins" [see Additional file 1]. The samples (ON, OFF, 10') in which each protein has been identified are specified together with the chromatographic method (anion-, cation-exchange). 131 proteins are found only in the sample ON, 104 in the sample OFF and 133 in the sample 10', indicating that every fraction contributes to the total identified proteins. The number of matched peptides per protein is shown. Single hit proteins resulted to be 193; corresponding to 24% of the total protein content.

To estimate the sensitivity of the chromatographic method coupled to nano-LC-MALDI analysis, we evaluated the number of proteins isolated by both resins (anion- and cation-exchange). The dynamic range of concentration covered by this approach was more than 7 orders of magnitude: the most concentrated was actin, while we identified the heat shock protein beta 1, a low concentrated marker [23]. After protein identification, we estimated the overlap percentage between anion-exchange and cation-exchange, which turned out to be 21.8%, suggesting that each resin tends to isolate different set of proteins. In particular, in the anion-exchange we isolated 480 unique proteins, while in the basic fraction we obtained 157 unique proteins. A total of 178 proteins resulted common to the two fractions. The proteins exclusively identified by the anion-exchanger represented almost 73% of all proteins, while those exclusively identified by the cation-exchanger were almost 47% of the total number of proteins identified. Even if the two pre-fractionation methods did not work identically, similar selection efficacies were demonstrated in the case of SCX and SAX columns for HPLC applications [24].

The identification of VSMC proteins from their peptide sequences was established with the Swiss-Prot database restricted to mammalian entries and the NCBI database restricted to Sus scrofa entries. A comparison was carried out between the two workflows on the basis of the number of proteins identified by using both databanks. Table 1 evidences the efficiency of MALDI analysis in mapping the VSMC proteome.

**Table 1:** Comparison of MALDI efficiency for the workflows with two databanks

workflow	MS Spectra^a)^	MSMS Spectra^b)^	Identified peptides	MSMS efficiency(%)^c)^	Proteins^d)^	peptides/proteins	Proteins by single peptide^e)^	DataBank^f)^
1 (Anion)	597	6868	3530	51%	658	5.4	141	SwissProt (Mammalia)
2 (Cation)	597	6212	2705	43%	335	8.1	139	SwissProt (Mammalia)
1 (Anion)	597	6868	4232	62%	900 (555Pred)	4.07	53	NCBI (Sus Scrofa)
2 (Cation)	597	6212	3317	54%	720 (467Pred)	4.07	34	NCBI (Sus Scrofa)

a) number of total MS spectra/workflow; b) number of total MSMS spectra/workflow; c) efficiency is determined dividing the number of identified peptides per MSMS spectra; d) number of unique identified proteins. "Pred" is an abbreviation for "Predicted" e) number of proteins identified by a single peptide as reported by GPS Explorer software; f) database chosen in protein identifications in GPS Explorer software.

Sensitivity is enhanced with respect to the classical 2D-PAGE without pre-fractionation steps [17] using pre-treatment with ion-exchange chromatography that avoids the mask effect due to the complexity of the whole cell protein extract.

The metric for the success of this study was to maximize sample information concerning identified peptides, number of proteins and minimum overlap between cation and anion exchange. The efficiency of the MALDI analysis strongly influences peptide identification and can be assessed by MSMS efficiency which compares the number of MSMS spectra with the number of identified peptides. We obtained a good performance in both cases and the comparison of MSMS efficacy in the two databanks reflects the strong dependence of sequence identification on the analysis settings used. While peptides identified by the SwissProt database all match for annotated protein sequences, peptides recognized by the NCBI databank only partially match for annotated proteins, more than half being predicted by genome annotations. The number of peptides for each protein (calculated as an average between the number of peptides and number of proteins) is lower in NCBI analysis than in SwissProt setting, but in NCBI analysis the number of proteins identified by a single peptide evidences a better distribution of the peptides within the proteins.

**Figure 3:**
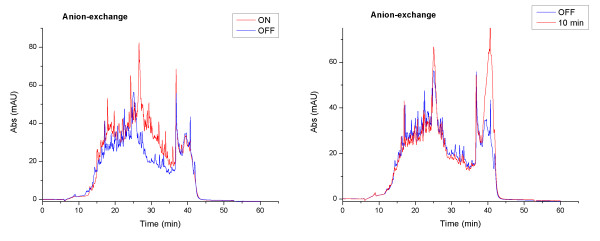
**HPLC UV Profile Overlap**. It shows the overlap of the UV profiles, measured by nano-HPLC at 214 nm wavelength of anion-exchange LC-runs in ON, OFF and 10 min "specific sample solutions"

After optimization of pre-fractionation and nano-LC-MALDI performance, we made a first attempt to compare the protein contents of activated ON-VSMC and quiescent OFF-VSMC obtained by anion-exchange. Significant differences between ON and OFF cells are made evident by simply comparing their UV profiles after HPLC separation of the peptide mixtures. Instead, the 10'-VSMC and OFF-VSMC UV profiles are strictly superimposed (Figure 3) as we expected, since in the early phase of activation post-translational modifications such as phosphorylation generally occur, while a differential protein expression is untimely [25]. For these reasons, to analyze the differences between activated and quiescent cells we concentrated our attention on ON and OFF samples.

**Figure 4:**
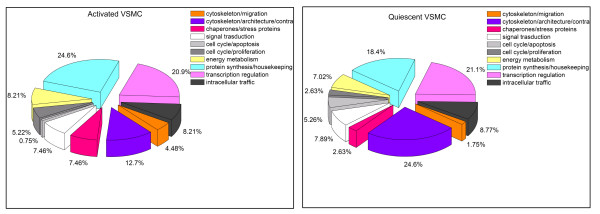
**Classification of identified proteins**. It shows identified protein distribution by selected functions respectively for ON and OFF cells.

The identified proteins in anion-exchange were grouped in functional classes and the differences between ON and OFF cells reported in figure 4. As a preliminary result, we can observe that major variations are related to cytoskeleton, chaperones, cell cycle, factors associated with protein synthesis and enzyme responsible of the energetic metabolism.

It is first interesting to observe how chaperones and enzymes involved in protein synthesis and energy metabolism are outnumbered in the activated cells. This phenotype is in fact characterized by the incentive of proliferation and migration and also by an impressive increase in protein synthesis [11,26]. It is encouraging to observe that proteins related to migration are more represented in activated cells (4% vs 2%), while the opposite is true for cytoskeleton elements implied with contraction (13% vs 24%). Moreover, proteins involved in the inhibition of cell proliferation or apoptosis are more numerous in quiescent VSMC, but proteins activating cell cycle progression are more represented in activated VSMC (5% vs 1%).

**Figure 5:**
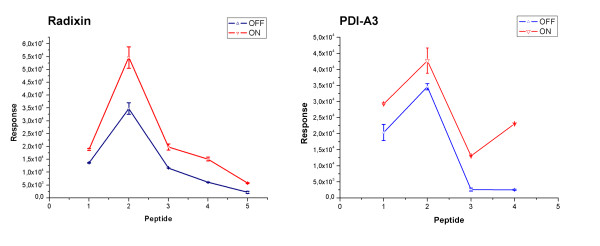
**Plot response/peptide m/z for two identified proteins in OFF and ON cells**. It shows the response of selected peptides (in terms of peptide area) in mass spectrometry recovered in OFF-VSMC (two technical replicates) and ON-VSMC (two technical replicates). The medium value of the response for every peptide and the error bars are reported. a) The selected peptides for Radixin are m/z 1) 987.5, .2)1104.5, 3) 1231.5, 4)1248.6, 5)2080.0 b) The selected peptides for PDI-A3 are m/z 1) 1172.5, 2)1191.6, 3)1359.6, 4)1664.7. Two interesting proteins are chosen and identified peptides are selected to analyze their general trend during the activation.

A preliminary comparative investigation was carried out to analyze the differential peptide and protein expression in the two different phenotypes. We used the aligned peak lists obtained by MarkerView [27] and two technical replicates for every sample (quiescent-OFF and activated-ON VSMC), with particular attention to some proteins clearly involved in functions undoubtedly stimulated by the growth factor. Identified peptides corresponding to a given protein were analyzed in order to follow the general trend. The result was encouraging since for the majority of the factors related to cytoskeleton remodelling responsible for cell motility an over-expression in activated VSMC was evidenced. We report the results obtained for two proteins, as examples (Figure 5).

VSMCs grown with 10% FBS for up to three days (ON) display completely different features and functions compared to the contractile-quiescent cells cultured in absence of serum (OFF); as a matter of fact, the existence of two distinctive phenotypes, one contractile and healthy, the other activated and pathological, has long been recognized.

## Conclusions

Ion-exchange chromatography is a suitable strategy aimed at simplifying the complexity of the cellular extract and at identifying a large number of proteins. The efficiency based on the ion-exchange chemical features was good if evaluated on the basis of the protein pI. The LC-MALDI approach was reproducible and sensitive since we were able to identify up to 815 proteins with a dynamic range of 7 orders of magnitude. Moreover, this approach could be easily integrated with other chromatographic media, more useful and selective for post-translational modifications and phospho-specific chromatographic methods such as IMAC, SIMAC or TiO_2 _useful to analyze the early events of VSMC activation. In our opinion, the large number of identified proteins and the promising quantitative reproducibility have made this approach a powerful method to analyze complex protein mixtures in a high throughput way and to obtain statistical data for the discovery of key factors involved in VSMC activation and to analyze a label-free differential protein expression.

## Methods

### Chemicals, standards and consumables

ACN and TFA used for LC-MS analysis or sample preparation were of HPLC quality (Sigma Aldrich, St Louis, USA); HPLC-grade water was prepared by a Milli-Q purification system (Millipore, Billerica, MA, USA); sequencing-grade trypsin for protein digestion was from Roche (Mannheim, Germany). BCA-Protein Assay for the determination of protein concentration was from Pierce (Rockford, IL, USA). All other chemicals used for sample preparation were reagent-grade from Sigma (St Louis, MO, USA).

### Isolation of VSMC and culture conditions

Coronaries were dissected from the myocardium of 8-month-old domestic crossbred pigs (Sus scrofa domestica). Medial VSMCs were isolated by enzymatic digestion and cultured according to the method described by Christen et al [19]. All experiments reported in this study were performed with VSMC at the sixth passage and, unless otherwise specified, from at least three different explants. The investigation was conformed to the Ministerial Decree to authorize testing n.06/2009-B of 26/01/2009.

### Protein extraction

VSMC pellets from about 10^7^ VSMCs were suspended in 500 μl of ice-cold lysis medium containing 50 mM Tris-HCl pH 8; 0.5% Triton X-100; 0.25% deoxycholic acid. Cells were sonicated (SoniPrep 150 MSE) 5 times (10 seconds sonication and 20 seconds rest in ice) and centrifuged (Centrifuge Eppendorf 5415R and a standard rotor F45-24-11, 11 mm diameter) at 10500 × g at 4°C for 30 min, gently vortexed and re-centrifuged for 15 min under the same conditions. The protein concentration was determined by BCA (bi-cinchoninic acid) and the protein samples were lyophilized.

### Protein fractionation

1.2 mg of each protein sample (OFF-quiescent cells, ON-activated cells, 10'-cells activated for 10 minutes with FBS) was re-suspended in 100 μl of water and split in two parts, one for anion-exchange chromatographic fractionation and the other for cation-exchange.

### Cation-exchange chromatographic fractionation

Cation-exchange columns were prepared using Mobicol column (Mo Bi Tec, Göttingen, Germany) filled with 250 μl of strong cation-exchanger resin (500 μl of slurry Unosphere S, Biorad). Each resin was centrifuged to remove the storage solution. They were washed with formic acid/NH_3 _pH 5.7 (binding buffer I) and centrifuged at 9300 × g for 30 sec; the wash was repeated twice. 450 μl of binding buffer I were added to the samples. The obtained solutions were loaded and fluttered up-down on the column for 15 min at 4°C. The columns were centrifuged at 9300 × g for 30 sec and washed with 150 μl of binding buffer I. The aspecific sample was discarded. Columns were washed with 0.5 ml of binding buffer I, vortexed and centrifuged twice at 9300 × g for 30 sec and the operation was repeated with 0.5 ml of water to favour pH change.

Elution of the specific fractions was carried out with 0.5 ml of elution buffer containing 5% NH_4_OH, 1 M NaCl, pH 11.8. The columns were fluttered up-down for 15 min at 4°C, centrifuged at 9300 × g for 30 sec and washed twice with 150 μl of elution buffer; the eluted was stored (named "specific" cationic sample solution). All samples were lyophilized.

### Anion-exchange chromatographic fractionation

Anion-exchange columns were prepared using Mobicol column (Mo Bi Tec, Göttingen, Germany) filled with 250 μl of strong anion-exchanger resin (500 μl of slurry Unosphere Q, Biorad). Each resin was centrifuged to remove the storage solution. They were washed with 200 mM Tris/HCl, pH 8.5 (binding buffer II) and centrifuged at 9300 × g for 30 sec; the wash was repeated twice. 450 μl of binding buffer II were added to the samples. The obtained solutions were loaded and fluttered up-down on the column for 15 min at 4°C. The columns were centrifuged at 9300 × g for 30 sec and washed with 150 μl of binding buffer II. The aspecific sample was discarded. Columns were washed with 0.5 ml of binding buffer II, vortexed and centrifuged twice at 9300 × g for 30 sec and the operation was repeated with 0.5 ml of water to favour pH change.

The columns were fluttered up-down for 15 min at 4°C, centrifuged at 9300 × g for 30 sec and washed twice with 150 μl of elution buffer (200 mM formic acid/NH_3 _pH = 2.5); the eluted was stored and named "specific" anionic sample solution. All samples were lyophilized.

### Reduction, alkylation and digestion

Lyophilized protein mixtures of samples were suspended in 250 μl of 20 mM ammonium hydrogen carbonate pH 8. Reduction was performed adding 25 μl of 50 mM DTT to each sample which was made to react at 80°C for 20 min. Samples were allowed to cool at room temperature and 12.5 μl 200 mM iodoacetamide were added for 30 min at 37°C. Digestion was performed adding 7.5 μl of trypsin solution (solution 0.25 mg/ml) at 37°C overnight.

### Peptide separation using Nano-HPLC

Chromatographic separation of peptides was performed using an Ultimate 3000 nano-HPLC system (LC Packings, DIONEX, Sunnyvale, USA). 37.5 μl of 0.2% TFA up to 150 μl with water was added to 50 μl of each sample and filtrated with 0.45 μm filter. The loading pump ensured pre-concentration of the sample in a μ-precolumn cartridge (PepMap-100 C18 5 μm 100A, 30 μm idx5 mm). Chromatographic separation of peptides was performed using a C18 PepMap-100 column (15 cm × 75 μm i.d., LC Packings DIONEX) and using a linear gradient of B, a mixture of acetonitrile/water (80:20) with 0.05% TFA, in solvent A, a mixture of water/acetonitrile (98:2) with 0.04% TFA as follows: from 10% to 45% of B in 30 min, switched to 100% buffer B for 10 min, followed by 20 min re-equilibration with buffer A at a constant flow rate of 0.3 μl/min. The column was directly coupled to a UV flow cell detector (214 nm-Ultimate DIONEX) at the exit of which the effluent was coaxially mixed with a solution of 2 mg/ml α-cyano-4-hydroxycinnamic acid MALDI matrix (α-CHCA) (Sigma-Aldrich, St Louis, MO, USA) prepared daily and delivered at a flow rate of 1.9 μl/min, then directed towards an on-line Probot (LC Packings DIONEX) plate-spotting system (MALDI plate containing 1664 total wells-ABSCIEX). The injection of each sample was replicated twice. Before and after sample analysis, two blank injections were performed to minimize carry-over. Each spot represented a 12-second "fraction" of the reverse phase gradient. The runs of each sample, each containing 200 spots, were distributed on the same MALDI plate.

### MALDI MS data acquisition

Spots representing the different chromatographic fractions were analyzed using a 4800 MALDI-TOF/TOF mass spectrometer (Applied Biosystems/MDS Sciex, Toronto, Canada), equipped with a laser emitting at λ = 355 nm with a repetition rate of 200 Hz. The mass spectrometer was controlled by the 4000 Series Explorer, version 3.5.2 program. For MS analyses, typically 2000 spectra were acquired for each spot in the reflector positive mode in the mass range of 650 to 4000 m/z, with 50 ppm mass tolerance (external and internal calibration). The spots of fractions eluted before 10 min and after 50 min were not analyzed for the sake of time, as preliminary investigations showed that they apparently contained very little peptide information.

### MS data processing and analysis

Peak lists obtained from Nano-LC-MALDI-TOF chromatograms were processed using MarkerView version 1.2 (Applied Biosystems/MDS Sciex, Toronto, Canada). Alignment was first performed with 2 min retention time and 0.2 amu mass tolerance for all technical replicates of OFF- and ON-VSMC. These settings were optimized values found to give a maximum number of common peaks between samples. Then, the data were scaled in intensity by normalization by total area sum using MarkerView. After alignment and normalization, the data were filtered out from MALDI residual α-CHCA matrix clusters. As most matrix clusters had masses below 700 m/z, filtration consisted in excluding all ions with m/z < 700. The aligned peak lists were used to plot profiles of interest peptides in the activated vs non-activated samples and to extract preliminary information on differential peptide/protein expression.

### MALDI MSMS data acquisition and protein identification

MS/MS data acquisition was performed on all runs in the MALDI for the best 15 precursor ions. MS/MS acquisitions were then carried out using air as collision gas at a pressure of ~3.0 × 10^6 ^torr and collision energy of 1 kV. Approximately 2000 spectra were added up for each spot. The peaks were de-isotoped and only those with s/n >5 were retained for interpretation.

MSMS data were pre-processed by the manufacturer computer program GPS Explorer version 3.6 (Applied Biosystems). Identification of pig proteins from their peptide sequences was established using MASCOT search engine version 2.1 (Matrix Science, Boston, MA) with the last updated version of Swiss-Prot protein database for Mammalia and UniGene database (NCBI) for Sus Scrofa (UniGene Built #37 Entries: 51,692). Methionine oxidation, Asn and Gln deamidation, Thr, Ser and Tyr phosphorylation and acetylation were selected as variable modifications. The tolerance for precursor ion and MS/MS fragment mass values was set at 50 ppm and 0.3 Da, respectively. Trypsin digestion and two possible missed cleavages were used. Only the 2 top-ranked peptide matches were taken into consideration for protein identification. Proteins with total ion score CI %> 97 (this parameter combines p-value for MSMS identification with MASCOT score) were accepted.

### Quantitative response and performance of MALDI-TOF/TOF analysis

Quantitative accuracy and reproducibility of MALDI-TOF/TOF were evaluated using an internal standard (ACTH 18-39 fragment, m/z 2465.16) added to the MALDI matrix and spotted along all the LC profile in known amount and in the same concentration in every spot. Three different concentrations (5-20 fmol/μl) were used to evaluate the linear increase of intensity with peptide concentration increase. Four replicates of each concentration were used to evaluate reproducibility.

## Competing interests

The authors declare that they have no competing interests.

## Authors' contributions

SR carried out HPLC separation, mass spectrometry analysis, data interpretation, data-base interrogation and manuscript drafting and revising. LC was involved in the interpretation of analytical data and protein function assignment. CB standardized ion-exchange chromatographic technique and HPLC-MALDI conditions. NU performed sample processing in proteomic fractionation and participated in VSMC culturing. LT was involved in sample processing and protein fractionation. CL performed protein extract preparations. MGT performed animal surgical interventions and coronary explanting according to bioethical guidelines. AC conceived the study, optimized cell culturing and cell activation conditions, coordinated cell explanting treatments and collaborated in all the biological aspects concerning compilation of the manuscript. All authors read and approved the final manuscript.

## Supplementary Material

Additional file 1 **Table-TotalUniqueProteins**. In the table total unique proteins are reported. The samples (ON, OFF, 10') in which each protein has been identified are specified, together with the chromatographic method (anion-, cation-exchange). Click here for file
