# Supplementary material for: A gel-free approach in vascular smooth muscle cell proteome: perspectives for a better insight into activation
Source: Proteome Sci. 2010 Mar 24;8:15. doi: 10.1186/1477-5956-8-15 (PMC2858725; doi:10.1186/1477-5956-8-15)
Supplement: Additional file 1 — Table-TotalUniqueProteins. In the table total unique proteins are reported. The samples (ON, OFF, 10') in which each protein has been identified are specified, together with the chromatographic method (anion-, cation-exchange). [file 1477-5956-8-15-S1.DOC]

| **PROTEIN NAME** | **Accession Number^a)^** | **Peptide Count^b)^** | **Anion-**  **Exchange^c)^** | **Cation-**  **Exchange^d)^** |
| --- | --- | --- | --- | --- |
| Filamin-A OS=Homo sapiens GN=FLNA PE=1 SV=4 | P21333 | 42 | ON-OFF-10 | ON-OFF-10 |
| Myosin-9 OS=Canis familiaris GN=MYH9 PE=2 SV=1 | Q258K2 | 37 | ON-OFF-10 | ON-OFF-10 |
| Fibronectin OS=Bos taurus GN=FN1 PE=1 SV=3 | P07589 | 36 | ON-OFF-10 | ON-OFF-10 |
| Vimentin OS=Homo sapiens GN=VIM PE=1 SV=4 | P08670 | 32 | ON-OFF | ON-OFF |
| Moesin OS=Sus scrofa GN=MSN PE=2 SV=3 | P26042 | 23 | ON-OFF-10 | ON-OFF-10 |
| Nuclease-sensitive element-binding protein 1 OS=Homo sapiens GN=YBX1 PE=1 SV=3 | P67809 | 18 | ON-OFF-10 | ON-OFF-10 |
| Vimentin (Fragments) OS=Sus scrofa GN=VIM PE=1 SV=1 | P02543 | 18 | ON-OFF-10 | ON-OFF-10 |
| Alpha-actinin-1 OS=Bos taurus GN=ACTN1 PE=2 SV=1 | Q3B7N2 | 17 | ON-OFF-10 | 10 |
| Actin, cytoplasmic 1 OS=Bos taurus GN=ACTB PE=1 SV=1 | P60712 | 16 | ON-OFF-10 | ON-OFF-10 |
| Actin, cytoplasmic 2 OS=Homo sapiens GN=ACTG1 PE=1 SV=1 | P63261 | 16 | ON-OFF-10 | ON-OFF-10 |
| Collagen alpha-1(I) chain OS=Homo sapiens GN=COL1A1 PE=1 SV=4 | P02452 | 16 | ON-OFF-10 | ON-OFF-10 |
| Heat shock cognate 71 kDa protein OS=Bos taurus GN=HSPA8 PE=1 SV=2 | P19120 | 15 | ON-OFF-10 |  |
| 40S ribosomal protein S19 OS=Homo sapiens GN=RPS19 PE=1 SV=2 | P39019 | 14 | ON-OFF-10 | ON-OFF-10 |
| Elongation factor 2 OS=Callithrix jacchus GN=EEF2 PE=2 SV=1 | A0SXL6 | 14 | ON-OFF-10 | ON-10 |
| Tropomyosin alpha-4 chain OS=Sus scrofa GN=TPM4 PE=1 SV=3 | P67937 | 14 | ON-OFF-10 | ON-OFF-10 |
| 78 kDa glucose-regulated protein OS=Bos taurus GN=HSPA5 PE=2 SV=1 | Q0VCX2 | 13 | ON-OFF-10 | ON-OFF-10 |
| Actin, alpha cardiac muscle 1 OS=Homo sapiens GN=ACTC1 PE=1 SV=1 | P68032 | 13 | ON-OFF-10 | OFF-10 |
| Actin, aortic smooth muscle OS=Homo sapiens GN=ACTA2 PE=1 SV=1 | P62736 | 13 | ON-OFF-10 |  |
| Heterogeneous nuclear ribonucleoprotein A1 OS=Bos taurus GN=HNRNPA1 PE=1 SV=2 | P09867 | 13 | ON-OFF-10 | ON-OFF-10 |
| Heterogeneous nuclear ribonucleoprotein A3 OS=Mus musculus GN=Hnrnpa3 PE=1 SV=1 | Q8BG05 | 13 | ON-OFF-10 | ON-OFF-10 |
| Lamin-A/C OS=Sus scrofa GN=LMNA PE=3 SV=1 | Q3ZD69 | 13 | ON-OFF-10 | 10 |
| Annexin A5 OS=Pan troglodytes GN=ANXA5 PE=2 SV=3 | Q5R1W0 | 12 | ON-OFF-10 |  |
| Heterogeneous nuclear ribonucleoproteins A2/B1 OS=Homo sapiens GN=HNRNPA2B1 PE=1 SV=2 | P22626 | 12 | ON-OFF-10 | ON-OFF-10 |
| Tropomyosin alpha-3 chain OS=Rattus norvegicus GN=Tpm3 PE=1 SV=2 | Q63610 | 12 | ON-OFF-10 | OFF-10 |
| ATP synthase subunit beta, mitochondrial OS=Bos taurus GN=ATP5B PE=1 SV=2 | P00829 | 11 | ON-OFF-10 |  |
| Ezrin OS=Homo sapiens GN=EZR PE=1 SV=4 | P15311 | 11 | ON-OFF-10 | OFF-10 |
| Splicing factor, arginine/serine-rich 1 OS=Homo sapiens GN=SFRS1 PE=1 SV=2 | Q07955 | 11 | ON-OFF-10 | OFF-10 |
| Tropomyosin beta chain OS=Homo sapiens GN=TPM2 PE=1 SV=1 | P07951 | 11 | ON-OFF-10 | OFF-10 |
| Vinculin OS=Rattus norvegicus GN=Vcl PE=1 SV=1 | P85972 | 11 | ON-OFF-10 |  |
| Alpha-actinin-4 OS=Mus musculus GN=Actn4 PE=1 SV=1 | P57780 | 10 | ON-OFF |  |
| Annexin A2 OS=Sus scrofa GN=ANXA2 PE=1 SV=4 | P19620 | 10 | ON-OFF-10 |  |
| High mobility group protein B1 OS=Callithrix jacchus GN=HMGB1 PE=3 SV=1 | B0CM99 | 10 | ON-OFF-10 | ON-OFF-10 |
| Keratin, type II cytoskeletal 1 OS=Homo sapiens GN=KRT1 PE=1 SV=5 | P04264 | 10 | ON-OFF-10 | 10 |
| Protein disulfide-isomerase A3 OS=Homo sapiens GN=PDIA3 PE=1 SV=4 | P30101 | 10 | ON-OFF-10 | OFF-10 |
| Radixin OS=Sus scrofa GN=RDX PE=2 SV=1 | P26044 | 10 | ON-OFF | ON-OFF-10 |
| Tropomyosin alpha-1 chain OS=Bos taurus GN=TPM1 PE=2 SV=1 | Q5KR49 | 10 | ON-OFF-10 | OFF-10 |
| Myosin light polypeptide 6 OS=Homo sapiens GN=MYL6 PE=1 SV=2 | P60660 | 9 | ON-OFF-10 | OFF-10 |
| Neuroblast differentiation-associated protein AHNAK OS=Homo sapiens GN=AHNAK PE=1 SV=2 | Q09666 | 9 | ON-OFF-10 | ON |
| Phosphoglycerate mutase 1 OS=Mus musculus GN=Pgam1 PE=1 SV=3 | Q9DBJ1 | 9 | ON-OFF-10 |  |
| Transgelin-2 OS=Bos taurus GN=TAGLN2 PE=2 SV=3 | Q5E9F5 | 9 | ON-OFF-10 | OFF |
| Tubulin beta-5 chain OS=Cricetulus griseus GN=TUBB5 PE=2 SV=1 | P69893 | 9 | ON-OFF-10 | 10 |
| 60S ribosomal protein L6 OS=Sus scrofa GN=RPL6 PE=2 SV=3 | Q2YGT9 | 8 | ON-OFF-10 | ON |
| Calmodulin OS=Homo sapiens GN=CALM1 PE=1 SV=2 | P62158 | 8 | ON-OFF-10 | ON |
| FK506-binding protein 10 OS=Bos taurus GN=FKBP10 PE=2 SV=1 | Q2HJ89 | 8 | ON-OFF-10 |  |
| Heterogeneous nuclear ribonucleoprotein K OS=Bos taurus GN=HNRNPK PE=2 SV=1 | Q3T0D0 | 8 | ON-OFF-10 | 10 |
| Histone H4 OS=Homo sapiens GN=HIST1H4A PE=1 SV=2 | P62805 | 8 | ON-OFF-10 | ON-OFF-10 |
| Myosin-10 OS=Bos taurus GN=MYH10 PE=2 SV=2 | Q27991 | 8 | ON-OFF-10 | OFF-10 |
| Rab GDP dissociation inhibitor beta OS=Sus scrofa GN=GDI2 PE=2 SV=1 | Q6Q7J2 | 8 | ON |  |
| Rho GDP-dissociation inhibitor 1 OS=Bos taurus GN=ARHGDIA PE=1 SV=3 | P19803 | 8 | ON-OFF-10 |  |
| Titin OS=Homo sapiens GN=TTN PE=1 SV=2 | Q8WZ42 | 8 | ON-OFF-10 | 10 |
| Transitional endoplasmic reticulum ATPase OS=Bos taurus GN=VCP PE=1 SV=1 | Q3ZBT1 | 8 | ON-OFF-10 |  |
| Tubulin beta-2A chain OS=Homo sapiens GN=TUBB2A PE=1 SV=1 | Q13885 | 8 | OFF | OFF |
| Alpha-actinin-2 OS=Bos taurus GN=ACTN2 PE=2 SV=1 | Q3ZC55 | 7 | ON |  |
| Alpha-enolase OS=Bos taurus GN=ENO1 PE=1 SV=4 | Q9XSJ4 | 7 | ON-OFF-10 |  |
| Beta-actin-like protein 2 OS=Mus musculus GN=Actbl2 PE=2 SV=1 | Q8BFZ3 | 7 | ON-OFF-10 | ON-OFF-10 |
| Collagen alpha-2(I) chain OS=Canis familiaris GN=COL1A2 PE=2 SV=2 | O46392 | 7 | OFF-10 | ON-OFF-10 |
| DNA-binding protein A OS=Homo sapiens GN=CSDA PE=1 SV=4 | P16989 | 7 | ON-OFF-10 | ON-OFF-10 |
| Eukaryotic translation initiation factor 5A-1 OS=Homo sapiens GN=EIF5A PE=1 SV=2 | P63241 | 7 | ON-OFF-10 | ON-OFF-10 |
| Filamin-C OS=Mus musculus GN=Flnc PE=1 SV=3 | Q8VHX6 | 7 | ON-OFF-10 | 10 |
| Galectin-1 OS=Sus scrofa GN=LGALS1 PE=2 SV=3 | Q49I35 | 7 | ON-OFF-10 | ON-10 |
| Golgi apparatus protein 1 OS=Mus musculus GN=Glg1 PE=1 SV=1 | Q61543 | 7 | ON-OFF-10 | 10 |
| Hepatoma-derived growth factor OS=Bos taurus GN=HDGF PE=2 SV=1 | Q9XSK7 | 7 | ON-OFF-10 | OFF-10 |
| Histone H2B type 2-B OS=Mus musculus GN=Hist2h2bb PE=1 SV=3 | Q64525 | 7 |  | ON-OFF |
| Histone H3.1 OS=Homo sapiens GN=HIST1H3A PE=1 SV=2 | P68431 | 7 | ON-10 | ON-OFF |
| Histone H3.2 OS=Homo sapiens GN=HIST2H3A PE=1 SV=3 | Q71DI3 | 7 | ON-OFF |  |
| Keratin, type I cytoskeletal 10 OS=Homo sapiens GN=KRT10 PE=1 SV=4 | P13645 | 7 | OFF-10 |  |
| Nucleolin OS=Rattus norvegicus GN=Ncl PE=1 SV=3 | P13383 | 7 | ON-OFF-10 | ON-OFF-10 |
| Polyadenylate-binding protein 1 OS=Homo sapiens GN=PABPC1 PE=1 SV=2 | P11940 | 7 | ON-OFF-10 |  |
| Splicing factor, arginine/serine-rich 2 OS=Homo sapiens GN=SFRS2 PE=1 SV=4 | Q01130 | 7 | ON-OFF-10 | ON-OFF-10 |
| Tubulin alpha-1B chain OS=Homo sapiens GN=TUBA1B PE=1 SV=1 | P68363 | 7 | ON-OFF | PFF |
| 40S ribosomal protein S15 OS=Mesocricetus auratus GN=RPS15 PE=2 SV=2 | P62842 | 6 | ON-OFF-10 | 10 |
| 40S ribosomal protein S18 OS=Canis familiaris GN=RPS18 PE=3 SV=3 | Q5TJE9 | 6 | ON-OFF-10 | ON-OFF-10 |
| ATP synthase subunit alpha heart isoform, mitochondrial OS=Bos taurus GN=ATP5A1 PE=1 SV=1 | P19483 | 6 | OFF-10 |  |
| Bullous pemphigoid antigen 1, isoforms 1/2/3/4 OS=Mus musculus GN=Dst PE=1 SV=1 | Q91ZU6 | 6 | ON-OFF | 10 |
| Caldesmon OS=Homo sapiens GN=CALD1 PE=1 SV=2 | Q05682 | 6 | ON-OFF-10 | ON-10 |
| Cationic trypsin OS=Bos taurus PE=1 SV=3 | P00760 | 6 | ON-OFF-10 | ON-OFF-10 |
| Chloride intracellular channel protein 1 OS=Homo sapiens GN=CLIC1 PE=1 SV=4 | O00299 | 6 | ON |  |
| Collagen alpha-1(III) chain OS=Homo sapiens GN=COL3A1 PE=1 SV=4 | P02461 | 6 | OFF-10 | OFF-10 |
| Eukaryotic translation initiation factor 2 subunit 2 OS=Oryctolagus cuniculus GN=EIF2S2 PE=1 SV=1 | P41035 | 6 | ON-OFF-10 | OFF-10 |
| FK506-binding protein 3 OS=Bos taurus GN=FKBP3 PE=1 SV=2 | P26884 | 6 | OFF | OFF-10 |
| Heterogeneous nuclear ribonucleoprotein A/B OS=Homo sapiens GN=HNRNPAB PE=1 SV=2 | Q99729 | 6 | ON-OFF-10 | OFF-10 |
| Heterogeneous nuclear ribonucleoprotein Q OS=Mus musculus GN=Syncrip PE=1 SV=2 | Q7TMK9 | 6 | ON | 10 |
| High mobility group protein B2 OS=Bos taurus GN=HMGB2 PE=1 SV=3 | P40673 | 6 | ON | OFF-10 |
| Histone H2B type 1-O OS=Homo sapiens GN=HIST1H2BO PE=1 SV=3 | P23527 | 6 |  | ON |
| Keratin, type I cytoskeletal 9 OS=Homo sapiens GN=KRT9 PE=1 SV=2 | P35527 | 6 | ON-OFF-10 |  |
| Peptidyl-prolyl cis-trans isomerase B OS=Bos taurus GN=PPIB PE=1 SV=3 | P80311 | 6 |  | OFF-10 |
| Putative annexin A2-like protein OS=Homo sapiens GN=ANXA2P2 PE=5 SV=2 | A6NMY6 | 6 | ON |  |
| Pyruvate kinase isozymes M1/M2 OS=Mus musculus GN=Pkm2 PE=1 SV=4 | P52480 | 6 | ON-OFF-10 |  |
| Serum albumin OS=Pongo abelii GN=ALB PE=2 SV=1 | Q5NVH5 | 6 | ON-10 | ON-OFF-10 |
| Stress-70 protein, mitochondrial OS=Cricetulus griseus GN=HSPA9 PE=2 SV=1 | O35501 | 6 | ON-OFF-10 |  |
| Talin-1 OS=Homo sapiens GN=TLN1 PE=1 SV=3 | Q9Y490 | 6 | ON-OFF-10 |  |
| Transcription factor A, mitochondrial OS=Sus scrofa GN=TFAM PE=2 SV=1 | Q5D144 | 6 |  | OFF-10 |
| Trifunctional enzyme subunit alpha, mitochondrial OS=Sus scrofa GN=HADHA PE=1 SV=1 | Q29554 | 6 | ON |  |
| Tubulin alpha-8 chain OS=Homo sapiens GN=TUBA8 PE=2 SV=1 | Q9NY65 | 6 | OFF |  |
| 40S ribosomal protein S29 OS=Homo sapiens GN=RPS29 PE=1 SV=2 | P62273 | 5 | OFF | ON-OFF-10 |
| 40S ribosomal protein SA OS=Cercopithecus aethiops GN=RPSA PE=2 SV=3 | Q2L9X0 | 5 | ON-OFF-10 |  |
| 60S ribosomal protein L15 OS=Homo sapiens GN=RPL15 PE=1 SV=2 | P61313 | 5 | ON-OFF-10 |  |
| 60S ribosomal protein L22 OS=Macaca fascicularis GN=RPL22 PE=2 SV=3 | Q4R5I3 | 5 | ON-OFF | OFF-10 |
| 60S ribosomal protein L7a OS=Homo sapiens GN=RPL7A PE=1 SV=2 | P62424 | 5 | ON-OFF |  |
| Abnormal spindle-like microcephaly-associated protein homolog OS=Macaca fascicularis GN=ASPM PE=2 SV=1 | P62291 | 5 | OFF-10 | OFF |
| ATP synthase subunit alpha liver isoform, mitochondrial (Fragment) OS=Sus scrofa GN=ATP5A2 PE=2 SV=1 | Q29596 | 5 | ON-OFF-10 | ON |
| ATP synthase subunit alpha, mitochondrial OS=Homo sapiens GN=ATP5A1 PE=1 SV=1 | P25705 | 5 | ON-10 | ON-OFF-10 |
| ATP synthase subunit e, mitochondrial OS=Sus scrofa GN=ATP5I PE=3 SV=4 | Q9MYT8 | 5 |  | ON-OFF |
| ATPase inhibitor, mitochondrial OS=Sus scrofa GN=ATPIF1 PE=3 SV=2 | Q29307 | 5 | ON-OFF-10 | ON-OFF-10 |
| Calumenin OS=Homo sapiens GN=CALU PE=1 SV=2 | O43852 | 5 | 10 | 10 |
| Desmin OS=Canis familiaris GN=DES PE=3 SV=3 | Q5XFN2 | 5 | ON | ON-10 |
| Glyceraldehyde-3-phosphate dehydrogenase OS=Sus scrofa GN=GAPDH PE=1 SV=4 | P00355 | 5 | ON-OFF-10 | ON-OFF-10 |
| Heat shock protein beta-1 OS=Sus scrofa GN=HSPB1 PE=2 SV=1 | Q5S1U1 | 5 | ON-OFF-10 | 10 |
| Heterogeneous nuclear ribonucleoprotein G OS=Pan troglodytes GN=RBMX PE=2 SV=1 | A5A6M3 | 5 | ON-OFF-10 |  |
| Heterogeneous nuclear ribonucleoprotein M OS=Rattus norvegicus GN=Hnrnpm PE=1 SV=4 | Q62826 | 5 | OFF-10 | OFF-10 |
| Heterogeneous nuclear ribonucleoprotein U OS=Homo sapiens GN=HNRNPU PE=1 SV=5 | Q00839 | 5 | ON-OFF-10 | ON-OFF-10 |
| Histone H2B type 1-K OS=Bos taurus GN=HIST1H2BK PE=1 SV=3 | Q2M2T1 | 5 |  | 10 |
| Histone H2B type 1-N OS=Bos taurus GN=HIST1H2BN PE=1 SV=3 | Q32L48 | 5 | ON-OFF-10 | OFF-10 |
| Myosin heavy chain, embryonic smooth muscle isoform (Fragment) OS=Oryctolagus cuniculus PE=2 SV=1 | Q99105 | 5 |  | OFF |
| Myosin regulatory light chain MRLC2 OS=Homo sapiens GN=MYLC2B PE=1 SV=2 | O14950 | 5 | ON-OFF-10 | ON-OFF-10 |
| Myosin regulatory light polypeptide 9 OS=Mus musculus GN=Myl9 PE=1 SV=3 | Q9CQ19 | 5 | ON-OFF-10 | ON-OFF-10 |
| Myosin-11 OS=Homo sapiens GN=MYH11 PE=1 SV=3 | P35749 | 5 | 10 | OFF-10 |
| Nebulin OS=Homo sapiens GN=NEB PE=1 SV=2 | P20929 | 5 | 10 |  |
| Nesprin-2 OS=Homo sapiens GN=SYNE2 PE=1 SV=3 | Q8WXH0 | 5 | OFF | ON |
| Neutral alpha-glucosidase AB OS=Sus scrofa GN=GANAB PE=1 SV=1 | P79403 | 5 | ON-OFF |  |
| Nuclear autoantigen Sp-100 OS=Homo sapiens GN=SP100 PE=1 SV=3 | P23497 | 5 | ON | OFF-10 |
| Nucleophosmin OS=Rattus norvegicus GN=Npm1 PE=1 SV=1 | P13084 | 5 | ON-OFF-10 | OFF-10 |
| Peroxiredoxin-1 OS=Bos taurus GN=PRDX1 PE=2 SV=1 | Q5E947 | 5 | ON-OFF-10 | 10 |
| Plasminogen activator inhibitor 1 RNA-binding protein OS=Mus musculus GN=Serbp1 PE=1 SV=2 | Q9CY58 | 5 | ON-OFF-10 |  |
| Pyruvate kinase isozyme M1 OS=Felis silvestris catus GN=PKM2 PE=1 SV=2 | P11979 | 5 | ON-OFF |  |
| Splicing factor, proline- and glutamine-rich OS=Homo sapiens GN=SFPQ PE=1 SV=2 | P23246 | 5 | ON-OFF-10 | OFF-10 |
| Staphylococcal nuclease domain-containing protein 1 OS=Bos taurus GN=SND1 PE=1 SV=1 | Q863B3 | 5 | ON-OFF-10 |  |
| T-complex protein 1 subunit beta OS=Bos taurus GN=CCT2 PE=2 SV=3 | Q3ZBH0 | 5 | ON-OFF-10 |  |
| Thioredoxin OS=Sus scrofa GN=TXN PE=1 SV=3 | P82460 | 5 | ON-OFF-10 | ON-OFF-10 |
| Tropomyosin alpha-1 chain OS=Bos taurus GN=TPM1 PE=2 SV=1 | Q5KR49 | 5 | ON |  |
| Tubulin alpha-8 chain OS=Homo sapiens GN=TUBA8 PE=2 SV=1 | Q9NY65 | 5 | ON |  |
| Tubulin beta-3 chain OS=Homo sapiens GN=TUBB3 PE=1 SV=2 | Q13509 | 5 | ON-OFF |  |
| 40S ribosomal protein S10 OS=Homo sapiens GN=RPS10 PE=1 SV=1 | P46783 | 4 | ON-OFF | 10 |
| 40S ribosomal protein S21 OS=Homo sapiens GN=RPS21 PE=1 SV=1 | P63220 | 4 | ON-OFF-10 | ON |
| 40S ribosomal protein S8 OS=Homo sapiens GN=RPS8 PE=1 SV=2 | P62241 | 4 |  | ON-OFF-10 |
| 60 kDa heat shock protein, mitochondrial OS=Cricetulus griseus GN=HSPD1 PE=2 SV=1 | P18687 | 4 | ON-OFF-10 |  |
| 60S ribosomal protein L13 OS=Homo sapiens GN=RPL13 PE=1 SV=4 | P26373 | 4 | ON-OFF-10 | ON-OFF-10 |
| 60S ribosomal protein L4 OS=Bos taurus GN=RPL4 PE=2 SV=3 | Q58DW0 | 4 | ON-OFF-10 | OFF-10 |
| 60S ribosomal protein L7 OS=Homo sapiens GN=RPL7 PE=1 SV=1 | P18124 | 4 | ON-OFF-10 | OFF-10 |
| Adenylyl cyclase-associated protein 1 OS=Macaca fascicularis GN=CAP1 PE=2 SV=3 | Q4R4I6 | 4 | ON-OFF-10 |  |
| Alpha-crystallin B chain OS=Mus musculus GN=Cryab PE=1 SV=2 | P23927 | 4 | ON-OFF-10 |  |
| Annexin A1 OS=Sus scrofa GN=ANXA1 PE=1 SV=3 | P19619 | 4 | ON-OFF-10 |  |
| Apolipoprotein B-100 OS=Rattus norvegicus GN=Apob PE=1 SV=1 | Q7TMA5 | 4 | OFF-10 | OFF |
| Apoptotic chromatin condensation inducer in the nucleus OS=Homo sapiens GN=ACIN1 PE=1 SV=1 | Q9UKV3 | 4 |  | ON-10 |
| ATP synthase-coupling factor 6, mitochondrial OS=Sus scrofa GN=ATP5J PE=1 SV=1 | P13618 | 4 | ON-OFF-10 |  |
| Calmodulin-like protein 3 OS=Rattus norvegicus GN=Calml3 PE=2 SV=1 | Q5U206 | 4 | ON-OFF-10 | ON |
| Centrosomal protein of 290 kDa OS=Homo sapiens GN=CEP290 PE=1 SV=2 | O15078 | 4 |  | 10 |
| Dystrophin-related protein 2 OS=Homo sapiens GN=DRP2 PE=2 SV=2 | Q13474 | 4 |  | ON |
| Elongation factor 1-gamma (Fragment) OS=Sus scrofa GN=EEF1G PE=2 SV=2 | Q29387 | 4 | ON-OFF |  |
| Elongation factor 1-gamma OS=Bos taurus GN=EEF1G PE=2 SV=1 | Q3SZV3 | 4 | ON-OFF-10 |  |
| Fascin OS=Homo sapiens GN=FSCN1 PE=1 SV=3 | Q16658 | 4 | ON |  |
| Filaggrin OS=Homo sapiens GN=FLG PE=1 SV=3 | P20930 | 4 | 10 |  |
| FUS-interacting serine-arginine-rich protein 1 OS=Mus musculus GN=Fusip1 PE=1 SV=2 | Q9R0U0 | 4 | ON-OFF-10 | OFF-10 |
| Heat shock 70 kDa protein 1L OS=Rattus norvegicus GN=Hspa1l PE=2 SV=2 | P55063 | 4 | ON-OFF |  |
| Heat shock 70 kDa protein 6 OS=Sus scrofa GN=HSPA6 PE=2 SV=1 | Q04967 | 4 | ON-OFF |  |
| Heterogeneous nuclear ribonucleoprotein D-like OS=Mus musculus GN=Hnrpdl PE=1 SV=1 | Q9Z130 | 4 |  | OFF-10 |
| Heterogeneous nuclear ribonucleoprotein F OS=Bos taurus GN=HNRNPF PE=2 SV=3 | Q5E9J1 | 4 | ON-OFF-10 |  |
| Histone H1.5 OS=Mus musculus GN=Hist1h1b PE=1 SV=2 | P43276 | 4 |  | ON-OFF |
| Histone H2A type 1-C OS=Rattus norvegicus PE=1 SV=2 | P0C169 | 4 | ON-OFF-10 | OFF-10 |
| Histone H2A type 1-D OS=Homo sapiens GN=HIST1H2AD PE=1 SV=2 | P20671 | 4 |  | 10 |
| Hypoxia up-regulated protein 1 OS=Cricetulus griseus GN=HYOU1 PE=2 SV=1 | Q60432 | 4 | ON-OFF-10 |  |
| Integrin beta-1 OS=Felis silvestris catus GN=ITGB1 PE=2 SV=1 | P53713 | 4 | OFF-10 |  |
| Interleukin enhancer-binding factor 3 OS=Mus musculus GN=Ilf3 PE=1 SV=2 | Q9Z1X4 | 4 | ON-OFF-10 | 10 |
| Intersectin-1 OS=Mus musculus GN=Itsn1 PE=1 SV=1 | Q9Z0R4 | 4 |  | OFF-10 |
| Lamin-A OS=Rattus norvegicus GN=Lmna PE=1 SV=1 | P48679 | 4 |  | OFF |
| Lamin-B1 OS=Mus musculus GN=Lmnb1 PE=1 SV=3 | P14733 | 4 | ON-OFF-10 |  |
| Macrophage-capping protein OS=Homo sapiens GN=CAPG PE=1 SV=1 | P40121 | 4 | ON-OFF-10 |  |
| Matrin-3 OS=Rattus norvegicus GN=Matr3 PE=1 SV=2 | P43244 | 4 | ON-OFF-10 |  |
| Microtubule-actin cross-linking factor 1 OS=Mus musculus GN=Macf1 PE=1 SV=1 | Q9QXZ0 | 4 |  | 10 |
| Microtubule-actin cross-linking factor 1, isoform 4 OS=Homo sapiens GN=MACF1 PE=1 SV=1 | Q96PK2 | 4 |  | 10 |
| Microtubule-actin cross-linking factor 1, isoforms 1/2/3/5 OS=Homo sapiens GN=MACF1 PE=1 SV=3 | Q9UPN3 | 4 |  | 10 |
| Nuclear ubiquitous casein and cyclin-dependent kinases substrate OS=Homo sapiens GN=NUCKS1 PE=1 SV=1 | Q9H1E3 | 4 | ON-OFF-10 |  |
| Nucleoside diphosphate kinase B OS=Sus scrofa GN=NME2 PE=2 SV=1 | Q2EN76 | 4 | ON-OFF |  |
| Paired mesoderm homeobox protein 1 OS=Homo sapiens GN=PRRX1 PE=2 SV=2 | P54821 | 4 |  | OFF-10 |
| Plastin-3 OS=Homo sapiens GN=PLS3 PE=1 SV=3 | P13797 | 4 | ON |  |
| Platelet-activating factor acetylhydrolase IB subunit beta OS=Homo sapiens GN=PAFAH1B2 PE=1 SV=1 | P68402 | 4 | ON |  |
| Polymerase I and transcript release factor OS=Mus musculus GN=Ptrf PE=1 SV=1 | O54724 | 4 | ON-OFF-10 | OFF-10 |
| Probable ATP-dependent RNA helicase DDX17 OS=Mus musculus GN=Ddx17 PE=2 SV=1 | Q501J6 | 4 | ON | 10 |
| Profilin-1 OS=Bos taurus GN=PFN1 PE=1 SV=2 | P02584 | 4 | ON-OFF-10 |  |
| Prohibitin-2 OS=Homo sapiens GN=PHB2 PE=1 SV=2 | Q99623 | 4 | ON-OFF-10 |  |
| Protein ARMET OS=Mus musculus GN=Armet PE=1 SV=1 | Q9CXI5 | 4 |  | ON-OFF-10 |
| Protein disulfide-isomerase OS=Homo sapiens GN=P4HB PE=1 SV=3 | P07237 | 4 | ON-OFF |  |
| Rab GDP dissociation inhibitor alpha OS=Canis familiaris GN=GDI1 PE=2 SV=1 | O97555 | 4 | ON |  |
| Ras GTPase-activating-like protein IQGAP1 OS=Mus musculus GN=Iqgap1 PE=1 SV=1 | Q9JKF1 | 4 | OFF-10 | ON-OFF-10 |
| Reticulocalbin-3 OS=Homo sapiens GN=RCN3 PE=1 SV=1 | Q96D15 | 4 | ON-OFF-10 |  |
| Serine/arginine repetitive matrix protein 2 OS=Mus musculus GN=Srrm2 PE=1 SV=2 | Q8BTI8 | 4 | ON |  |
| Small nuclear ribonucleoprotein Sm D2 OS=Homo sapiens GN=SNRPD2 PE=1 SV=1 | P62316 | 4 | ON-OFF-10 |  |
| Splicing factor, arginine/serine-rich 2B OS=Homo sapiens GN=SFRS2B PE=1 SV=1 | Q9BRL6 | 4 |  | OFF-10 |
| Splicing factor, arginine/serine-rich 3 OS=Homo sapiens GN=SFRS3 PE=1 SV=1 | P84103 | 4 | ON-OFF-10 | 10 |
| Splicing factor, arginine/serine-rich 7 OS=Homo sapiens GN=SFRS7 PE=1 SV=1 | Q16629 | 4 | ON-OFF-10 | OFF-10 |
| Thrombospondin-1 OS=Bos taurus GN=THBS1 PE=2 SV=2 | Q28178 | 4 | ON-OFF-10 | OFF-10 |
| Transaldolase OS=Bos taurus GN=TALDO1 PE=2 SV=1 | Q2TBL6 | 4 | ON-OFF-10 |  |
| Triosephosphate isomerase OS=Rattus norvegicus GN=Tpi1 PE=1 SV=2 | P48500 | 4 | ON-OFF-10 |  |
| Tubulin beta-6 chain OS=Mus musculus GN=Tubb6 PE=1 SV=1 | Q922F4 | 4 | ON-OFF-10 | 10 |
| Tyrosine-protein phosphatase non-receptor type 14 OS=Mus musculus GN=Ptpn14 PE=1 SV=1 | Q62130 | 4 | 10 |  |
| Ubiquitin carboxyl-terminal hydrolase isozyme L1 OS=Sus scrofa GN=UCHL1 PE=2 SV=1 | Q6SEG5 | 4 | ON-OFF-10 |  |
| Uncharacterized protein C12orf35 homolog OS=Mus musculus GN=Kiaa1551 PE=2 SV=2 | Q5DTW7 | 4 | 10 |  |
| UV excision repair protein RAD23 homolog B OS=Mus musculus GN=Rad23b PE=1 SV=1 | P54728 | 4 | ON-OFF-10 |  |
| 10 kDa heat shock protein, mitochondrial OS=Homo sapiens GN=HSPE1 PE=1 SV=2 | P61604 | 3 | ON | OFF-10 |
| 14-3-3 protein zeta/delta OS=Homo sapiens GN=YWHAZ PE=1 SV=1 | P63104 | 3 | ON-OFF-10 |  |
| 28 kDa heat- and acid-stable phosphoprotein OS=Homo sapiens GN=PDAP1 PE=1 SV=1 | Q13442 | 3 |  | 10 |
| 40S ribosomal protein S3a (Fragment) OS=Felis silvestris catus GN=RPS3A PE=2 SV=1 | P61246 | 3 | ON-OFF-10 |  |
| 40S ribosomal protein S4, X isoform OS=Felis silvestris catus GN=RPS4X PE=2 SV=2 | P62705 | 3 | ON-OFF |  |
| 40S ribosomal protein S4, Y isoform 1 OS=Macaca fuscata fuscata GN=RPS4Y1 PE=2 SV=3 | P79183 | 3 | ON |  |
| 40S ribosomal protein S9 OS=Homo sapiens GN=RPS9 PE=1 SV=3 | P46781 | 3 | ON |  |
| 5-azacytidine-induced protein 1 OS=Mus musculus GN=Azi1 PE=1 SV=1 | Q62036 | 3 | 10 |  |
| 60S ribosomal protein L10a (Fragment) OS=Sus scrofa GN=RPL10A PE=2 SV=3 | P53027 | 3 | ON-OFF | ON-OFF |
| 60S ribosomal protein L10a OS=Homo sapiens GN=RPL10A PE=1 SV=2 | P62906 | 3 | ON | ON-OFF |
| 60S ribosomal protein L17 OS=Felis silvestris catus GN=RPL17 PE=2 SV=3 | Q5XTY7 | 3 | OFF-10 | ON-OFF-10 |
| 60S ribosomal protein L19 OS=Homo sapiens GN=RPL19 PE=1 SV=1 | P84098 | 3 | ON | ON-OFF-10 |
| 60S ribosomal protein L26 OS=Homo sapiens GN=RPL26 PE=1 SV=1 | P61254 | 3 | ON-OFF | ON-OFF-10 |
| 60S ribosomal protein L26-like 1 OS=Homo sapiens GN=RPL26L1 PE=2 SV=1 | Q9UNX3 | 3 | ON |  |
| 60S ribosomal protein L3 OS=Mus musculus GN=Rpl3 PE=2 SV=2 | P27659 | 3 | ON | ON |
| 60S ribosomal protein L37 OS=Homo sapiens GN=RPL37 PE=2 SV=2 | P61927 | 3 |  | ON-OFF |
| 60S ribosomal protein L5 OS=Bos taurus GN=RPL5 PE=2 SV=3 | Q58DW5 | 3 | ON-OFF-10 | OFF-10 |
| Absent in melanoma 1 protein OS=Homo sapiens GN=AIM1 PE=1 SV=3 | Q9Y4K1 | 3 | 10 |  |
| Adenomatous polyposis coli protein OS=Mus musculus GN=Apc PE=1 SV=1 | Q61315 | 3 | OFF |  |
| Alpha-2-macroglobulin receptor-associated protein OS=Rattus norvegicus GN=Lrpap1 PE=1 SV=2 | Q99068 | 3 |  | OFF |
| AP-2 complex subunit mu-1 OS=Homo sapiens GN=AP2M1 PE=1 SV=2 | Q96CW1 | 3 | ON-OFF |  |
| Aspartate aminotransferase, cytoplasmic OS=Sus scrofa GN=GOT1 PE=1 SV=3 | P00503 | 3 | ON |  |
| AT-hook-containing transcription factor 1 OS=Mus musculus GN=Ahctf1 PE=1 SV=1 | Q8CJF7 | 3 | OFF |  |
| ATP-binding cassette sub-family F member 1 OS=Sus scrofa GN=ABCF1 PE=3 SV=1 | Q767L0 | 3 |  | OFF-10 |
| ATP-dependent RNA helicase A OS=Bos taurus GN=DHX9 PE=2 SV=1 | Q28141 | 3 | ON |  |
| Baculoviral IAP repeat-containing protein 6 OS=Homo sapiens GN=BIRC6 PE=1 SV=1 | Q9NR09 | 3 | ON |  |
| Bcl-2-associated transcription factor 1 OS=Homo sapiens GN=BCLAF1 PE=1 SV=2 | Q9NYF8 | 3 | OFF-10 |  |
| Beta-enolase OS=Mus musculus GN=Eno3 PE=1 SV=3 | P21550 | 3 |  | ON |
| Breast cancer type 2 susceptibility protein homolog OS=Rattus norvegicus GN=Brca2 PE=1 SV=1 | O35923 | 3 | 10 |  |
| Calponin-1 OS=Sus scrofa GN=CNN1 PE=2 SV=1 | Q08092 | 3 | ON | 10 |
| Calreticulin OS=Bos taurus GN=CALR PE=1 SV=2 | P52193 | 3 | ON-OFF |  |
| Cathepsin K OS=Sus scrofa GN=CTSK PE=2 SV=1 | Q9GLE3 | 3 | ON-OFF-10 | 10 |
| CD109 antigen OS=Mus musculus GN=Cd109 PE=2 SV=1 | Q8R422 | 3 |  | OFF |
| Centrosome-associated protein 350 OS=Homo sapiens GN=CEP350 PE=1 SV=1 | Q5VT06 | 3 | OFF |  |
| Coactosin-like protein OS=Bos taurus GN=COTL1 PE=2 SV=3 | Q2HJ57 | 3 | ON-OFF |  |
| Cofilin-1 OS=Macaca fascicularis GN=CFL1 PE=2 SV=3 | Q4R5C0 | 3 | ON-OFF-10 | OFF-10 |
| Coiled-coil domain-containing protein 91 OS=Homo sapiens GN=CCDC91 PE=1 SV=1 | Q7Z6B0 | 3 | 10 |  |
| Collagen alpha-1(VI) chain OS=Homo sapiens GN=COL6A1 PE=1 SV=3 | P12109 | 3 | ON-OFF-10 |  |
| Collagen alpha-1(XIX) chain OS=Homo sapiens GN=COL19A1 PE=1 SV=3 | Q14993 | 3 |  |  |
| CREB-binding protein OS=Mus musculus GN=Crebbp PE=1 SV=2 | P45481 | 3 | 10 |  |
| Cubilin OS=Canis familiaris GN=CUBN PE=1 SV=1 | Q9TU53 | 3 | ON-10 | 10 |
| Dihydrolipoyllysine-residue succinyltransferase component of 2-oxoglutarate dehydrogenase complex, mitochondrial OS=Sus scrofa GN=DLST PE=1 SV=1 | Q9N0F1 | 3 | ON-OFF-10 |  |
| Drebrin-like protein OS=Mus musculus GN=Dbnl PE=1 SV=2 | Q62418 | 3 | ON-OFF-10 |  |
| Dual oxidase 1 OS=Canis familiaris GN=DUOX1 PE=1 SV=1 | Q9MZF4 | 3 |  | OFF |
| Dual specificity testis-specific protein kinase 2 OS=Rattus norvegicus GN=Tesk2 PE=2 SV=1 | Q924U5 | 3 |  | 10 |
| E3 ubiquitin-protein ligase HUWE1 OS=Homo sapiens GN=HUWE1 PE=1 SV=3 | Q7Z6Z7 | 3 | 10 |  |
| Elongation factor 1-alpha 1 OS=Cricetulus griseus GN=EEF1A1 PE=2 SV=1 | P62629 | 3 | ON-OFF | ON-OFF-10 |
| EMILIN-1 OS=Homo sapiens GN=EMILIN1 PE=1 SV=2 | Q9Y6C2 | 3 | OFF-10 |  |
| Enamelin OS=Sus scrofa GN=ENAM PE=1 SV=1 | O97939 | 3 |  | 10 |
| Endoplasmin OS=Bos taurus GN=HSP90B1 PE=2 SV=1 | Q95M18 | 3 | ON-OFF |  |
| Enhancer of rudimentary homolog OS=Homo sapiens GN=ERH PE=1 SV=1 | P84090 | 3 | ON-OFF-10 |  |
| Eukaryotic translation initiation factor 1A, X-chromosomal OS=Mus musculus GN=Eif1ay PE=2 SV=3 | Q8BMJ3 | 3 | ON-OFF | OFF-10 |
| Eukaryotic translation initiation factor 3 subunit A OS=Homo sapiens GN=EIF3A PE=1 SV=1 | Q14152 | 3 | ON-OFF-10 | ON-10 |
| Eukaryotic translation initiation factor 4B OS=Homo sapiens GN=EIF4B PE=1 SV=2 | P23588 | 3 | ON-OFF-10 |  |
| Eukaryotic translation initiation factor 4H OS=Homo sapiens GN=EIF4H PE=1 SV=5 | Q15056 | 3 | ON-OFF-10 |  |
| Exocyst complex component 3-like protein 2 OS=Homo sapiens GN=EXOC3L2 PE=2 SV=1 | Q2M3D2 | 3 | OFF |  |
| Far upstream element-binding protein 1 OS=Homo sapiens GN=FUBP1 PE=1 SV=3 | Q96AE4 | 3 | ON-10 |  |
| Fibroblast growth factor 22 OS=Homo sapiens GN=FGF22 PE=1 SV=1 | Q9HCT0 | 3 |  | 10 |
| Filamin-B OS=Homo sapiens GN=FLNB PE=1 SV=1 | O75369 | 3 | ON-OFF-10 |  |
| Glial fibrillary acidic protein OS=Bos taurus GN=GFAP PE=2 SV=2 | Q28115 | 3 | 10 |  |
| Glutamate dehydrogenase 2, mitochondrial OS=Pan troglodytes GN=GLUD2 PE=3 SV=1 | Q64HZ8 | 3 |  | OFF-10 |
| Golgi integral membrane protein 4 OS=Homo sapiens GN=GOLIM4 PE=1 SV=1 | O00461 | 3 | OFF |  |
| Golgin subfamily A member 5 OS=Mus musculus GN=Golga5 PE=1 SV=2 | Q9QYE6 | 3 |  | OFF |
| Hepatocyte growth factor-regulated tyrosine kinase substrate OS=Rattus norvegicus GN=Hgs PE=1 SV=1 | Q9JJ50 | 3 | 10 |  |
| Hepatoma-derived growth factor-related protein 2 OS=Mus musculus GN=Hdgfrp2 PE=1 SV=1 | Q3UMU9 | 3 |  | OFF |
| Hepatoma-derived growth factor-related protein 3 OS=Rattus norvegicus GN=Hdgfrp3 PE=2 SV=1 | Q923W4 | 3 | OFF | OFF |
| Heterogeneous nuclear ribonucleoprotein A0 OS=Homo sapiens GN=HNRNPA0 PE=1 SV=1 | Q13151 | 3 | ON-OFF-10 | 10 |
| Heterogeneous nuclear ribonucleoprotein D0 OS=Mus musculus GN=Hnrnpd PE=1 SV=2 | Q60668 | 3 | ON-OFF-10 | 10 |
| Heterogeneous nuclear ribonucleoprotein H OS=Homo sapiens GN=HNRNPH1 PE=1 SV=4 | P31943 | 3 | ON-OFF-10 | ON |
| Heterogeneous nuclear ribonucleoprotein R OS=Homo sapiens GN=HNRNPR PE=1 SV=1 | O43390 | 3 | ON | OFF-10 |
| High mobility group protein B3 OS=Bos taurus GN=HMGB3 PE=2 SV=2 | Q32L31 | 3 |  | ON-OFF-10 |
| Histone H2A.V OS=Homo sapiens GN=H2AFV PE=1 SV=3 | Q71UI9 | 3 |  | ON-10 |
| Histone H2A.x OS=Homo sapiens GN=H2AFX PE=1 SV=2 | P16104 | 3 | ON-OFF |  |
| Histone H2B type F-S OS=Homo sapiens GN=H2BFS PE=1 SV=2 | P57053 | 3 | ON-OFF-10 |  |
| Histone-lysine N-methyltransferase MLL2 OS=Homo sapiens GN=MLL2 PE=1 SV=1 | O14686 | 3 | ON |  |
| Homeobox protein aristaless-like 4 OS=Homo sapiens GN=ALX4 PE=1 SV=1 | Q9H161 | 3 |  | OFF |
| Homeobox protein ARX OS=Mus musculus GN=Arx PE=2 SV=3 | O35085 | 3 |  | OFF |
| Hornerin OS=Homo sapiens GN=HRNR PE=1 SV=2 | Q86YZ3 | 3 | 10 |  |
| Keratin, type II cuticular Hb4 OS=Homo sapiens GN=KRT84 PE=2 SV=1 | Q9NSB2 | 3 | ON |  |
| Keratin, type II cytoskeletal 1b OS=Rattus norvegicus GN=Krt77 PE=2 SV=1 | Q6IG01 | 3 | OFF-10 |  |
| Keratin, type II cytoskeletal 2 epidermal OS=Homo sapiens GN=KRT2 PE=1 SV=1 | P35908 | 3 | 10 |  |
| Keratin, type II cytoskeletal 4 OS=Rattus norvegicus GN=Krt4 PE=2 SV=1 | Q6IG00 | 3 | 10 |  |
| Keratin, type II cytoskeletal 5 OS=Rattus norvegicus GN=Krt5 PE=1 SV=1 | Q6P6Q2 | 3 | ON-OFF-10 | 10 |
| Keratin, type II cytoskeletal 6A OS=Rattus norvegicus GN=Krt6a PE=1 SV=1 | Q4FZU2 | 3 | OFF-10 |  |
| Keratin, type II cytoskeletal 7 OS=Bos taurus GN=KRT7 PE=2 SV=1 | Q29S21 | 3 | ON-OFF | 10 |
| Keratin, type II cytoskeletal 79 OS=Mus musculus GN=Krt79 PE=2 SV=2 | Q8VED5 | 3 | ON-OFF-10 | OFF-10 |
| Keratin, type II cytoskeletal 8 OS=Homo sapiens GN=KRT8 PE=1 SV=7 | P05787 | 3 | OFF-10 |  |
| Kinesin heavy chain isoform 5A OS=Pongo abelii GN=KIF5A PE=2 SV=1 | Q5R9K7 | 3 | 10 |  |
| Kinesin-like protein KIF1A OS=Homo sapiens GN=KIF1A PE=2 SV=2 | Q12756 | 3 |  | OFF-10 |
| L-lactate dehydrogenase A chain OS=Sus scrofa GN=LDHA PE=1 SV=3 | P00339 | 3 | ON-OFF |  |
| Laminin subunit alpha-5 OS=Mus musculus GN=Lama5 PE=1 SV=3 | Q61001 | 3 | ON |  |
| Leucine-rich repeat and calponin homology domain-containing protein 3 OS=Mus musculus GN=Lrch3 PE=2 SV=2 | Q8BVU0 | 3 | OFF |  |
| Leucine-rich repeat-containing protein 59 OS=Homo sapiens GN=LRRC59 PE=1 SV=1 | Q96AG4 | 3 |  | OFF-10 |
| LIM and SH3 domain protein 1 OS=Rattus norvegicus GN=Lasp1 PE=1 SV=1 | Q99MZ8 | 3 | ON-10 |  |
| Lupus La protein OS=Homo sapiens GN=SSB PE=1 SV=2 | P05455 | 3 | ON |  |
| Mesoderm development candidate 2 OS=Bos taurus GN=MESDC2 PE=2 SV=1 | Q3T0U1 | 3 | 10 |  |
| Midasin OS=Homo sapiens GN=MDN1 PE=1 SV=2 | Q9NU22 | 3 | OFF |  |
| Mitochondrial tumor suppressor 1 homolog OS=Mus musculus GN=Mtus1 PE=1 SV=2 | Q5HZI1 | 3 |  | ON |
| Myomegalin OS=Homo sapiens GN=PDE4DIP PE=2 SV=1 | Q5VU43 | 3 | OFF | 10 |
| Myomesin-1 OS=Mus musculus GN=Myom1 PE=1 SV=1 | Q62234 | 3 |  | OFF |
| Myosin light chain kinase, smooth muscle OS=Mus musculus GN=Mylk PE=1 SV=3 | Q6PDN3 | 3 |  | ON |
| Neurofilament heavy polypeptide OS=Rattus norvegicus GN=Nefh PE=1 SV=4 | P16884 | 3 | OFF | 10 |
| Nuclear migration protein nudC OS=Rattus norvegicus GN=Nudc PE=1 SV=1 | Q63525 | 3 | ON |  |
| Nuclear receptor corepressor 1 OS=Homo sapiens GN=NCOR1 PE=1 SV=2 | O75376 | 3 | ON |  |
| Nuclear receptor corepressor 2 OS=Mus musculus GN=Ncor2 PE=1 SV=2 | Q9WU42 | 3 | ON |  |
| PC4 and SFRS1-interacting protein OS=Homo sapiens GN=PSIP1 PE=1 SV=1 | O75475 | 3 | OFF-10 | OFF-10 |
| Peptidyl-prolyl cis-trans isomerase A OS=Bos taurus GN=PPIA PE=1 SV=2 | P62935 | 3 | ON-OFF | 10 |
| Pericentriolar material 1 protein OS=Mus musculus GN=Pcm1 PE=1 SV=1 | Q9R0L6 | 3 |  | OFF-10 |
| Peripherin OS=Rattus norvegicus GN=Prph PE=1 SV=1 | P21807 | 3 |  | 10 |
| Peroxiredoxin-6 OS=Sus scrofa GN=PRDX6 PE=2 SV=3 | Q9TSX9 | 3 | ON-OFF-10 |  |
| Peroxisome assembly factor 2 OS=Homo sapiens GN=PEX6 PE=1 SV=2 | Q13608 | 3 | 10 |  |
| Plectin-1 OS=Homo sapiens GN=PLEC1 PE=1 SV=3 | Q15149 | 3 | ON-OFF-10 |  |
| Polyadenylate-binding protein 4 OS=Homo sapiens GN=PABPC4 PE=1 SV=1 | Q13310 | 3 | ON-OFF |  |
| Probable E3 ubiquitin-protein ligase TRIP12 OS=Homo sapiens GN=TRIP12 PE=1 SV=1 | Q14669 | 3 |  | ON |
| Probable RNA-binding protein 25 OS=Homo sapiens GN=RBM25 PE=1 SV=2 | P49756 | 3 |  | ON-OFF-10 |
| Proliferation-associated protein 2G4 OS=Mus musculus GN=Pa2g4 PE=1 SV=3 | P50580 | 3 | ON | 10 |
| Prolyl 4-hydroxylase subunit alpha-1 OS=Rattus norvegicus GN=P4ha1 PE=2 SV=2 | P54001 | 3 | ON-OFF |  |
| Proteasome activator complex subunit 1 OS=Bos taurus GN=PSME1 PE=1 SV=1 | Q4U5R3 | 3 | ON-OFF-10 |  |
| Proteasome subunit beta type-1 OS=Homo sapiens GN=PSMB1 PE=1 SV=2 | P20618 | 3 | ON-OFF |  |
| Protein canopy homolog 2 OS=Homo sapiens GN=CNPY2 PE=1 SV=1 | Q9Y2B0 | 3 | ON-OFF-10 |  |
| Protein dpy-30 homolog OS=Homo sapiens GN=DPY30 PE=1 SV=1 | Q9C005 | 3 | ON-OFF-10 |  |
| Protein phosphatase 1 regulatory subunit 12A OS=Homo sapiens GN=PPP1R12A PE=1 SV=1 | O14974 | 3 | OFF | 10 |
| Putative uncharacterized protein LOC338809 OS=Homo sapiens PE=2 SV=1 | Q32Q52 | 3 |  | ON |
| Ras GTPase-activating protein-binding protein 1 OS=Bos taurus GN=G3BP PE=2 SV=1 | Q32LC7 | 3 | ON-OFF-10 |  |
| Replicase polyprotein 1ab OS=Human SARS coronavirus GN=rep PE=1 SV=1 | P0C6X7 | 3 |  | OFF |
| Ribosome-binding protein 1 OS=Mus musculus GN=Rrbp1 PE=2 SV=2 | Q99PL5 | 3 | ON-OFF-10 | OFF-10 |
| RNA and export factor-binding protein 2 OS=Mus musculus GN=Refbp2 PE=1 SV=1 | Q9JJW6 | 3 | 10 |  |
| Rootletin OS=Homo sapiens GN=CROCC PE=1 SV=1 | Q5TZA2 | 3 |  | 10 |
| Septin-7 OS=Homo sapiens GN=SEPT7 PE=1 SV=2 | Q16181 | 3 | ON |  |
| Serine hydroxymethyltransferase, mitochondrial OS=Bos taurus GN=SHMT2 PE=2 SV=1 | Q3SZ20 | 3 | ON-OFF-10 | ON |
| Serpin H1 OS=Pongo abelii GN=SERPINH1 PE=2 SV=1 | Q5RBS3 | 3 | ON-OFF-10 | OFF-10 |
| Small nuclear ribonucleoprotein Sm D3 OS=Mus musculus GN=Snrpd3 PE=2 SV=1 | P62320 | 3 | ON-OFF-10 |  |
| Sorting nexin-18 OS=Homo sapiens GN=SNX18 PE=1 SV=1 | Q96RF0 | 3 | ON-OFF-10 |  |
| Spectrin alpha chain, brain OS=Rattus norvegicus GN=Sptan1 PE=1 SV=2 | P16086 | 3 | ON-OFF |  |
| Spectrin beta chain, brain 1 OS=Homo sapiens GN=SPTBN1 PE=1 SV=2 | Q01082 | 3 |  | OFF-10 |
| Spectrin beta chain, brain 2 OS=Rattus norvegicus GN=Sptbn2 PE=1 SV=2 | Q9QWN8 | 3 | ON |  |
| Spectrin beta chain, brain 4 OS=Homo sapiens GN=SPTBN5 PE=2 SV=1 | Q9NRC6 | 3 | ON |  |
| Splicing factor 1 OS=Homo sapiens GN=SF1 PE=1 SV=4 | Q15637 | 3 | ON-OFF-10 |  |
| Splicing factor, arginine/serine-rich 10 OS=Homo sapiens GN=SFRS10 PE=1 SV=1 | P62995 | 3 | ON-OFF-10 | ON-OFF-10 |
| Src substrate cortactin OS=Mus musculus GN=Cttn PE=1 SV=1 | Q60598 | 3 | ON-OFF-10 |  |
| SWI/SNF-related matrix-associated actin-dependent regulator of chromatin subfamily A containing DEAD/H box 1 OS=Mus musculus GN=Smarcad1 PE=1 SV=2 | Q04692 | 3 | OFF-10 | ON |
| Synembryn-A OS=Mus musculus GN=Ric8a PE=1 SV=2 | Q3TIR3 | 3 | 10 |  |
| T-complex protein 1 subunit epsilon OS=Macaca fascicularis GN=CCT5 PE=2 SV=1 | Q4R6V2 | 3 | ON |  |
| Teneurin-3 OS=Mus musculus GN=Odz3 PE=2 SV=1 | Q9WTS6 | 3 | OFF |  |
| TFIIH basal transcription factor complex helicase subunit OS=Bos taurus GN=ERCC2 PE=2 SV=1 | A6QLJ0 | 3 | ON |  |
| THO complex subunit 4 OS=Mus musculus GN=Thoc4 PE=1 SV=3 | O08583 | 3 | ON-OFF-10 |  |
| Thyroid hormone receptor-associated protein 3 OS=Homo sapiens GN=THRAP3 PE=1 SV=2 | Q9Y2W1 | 3 | ON-10 |  |
| Tight junction protein ZO-1 OS=Canis familiaris GN=TJP1 PE=1 SV=1 | O97758 | 3 | 10 |  |
| Transcription elongation regulator 1 OS=Mus musculus GN=Tcerg1 PE=1 SV=2 | Q8CGF7 | 3 |  | ON-OFF-10 |
| Transcription factor BTF3 OS=Homo sapiens GN=BTF3 PE=1 SV=1 | P20290 | 3 |  | ON-10 |
| Transferrin receptor protein 1 OS=Canis familiaris GN=TFRC PE=2 SV=1 | Q9GLD3 | 3 |  | 10 |
| Transgelin OS=Bos taurus GN=TAGLN PE=1 SV=4 | Q9TS87 | 3 | ON-OFF-10 |  |
| Transient receptor potential cation channel subfamily M member 6 OS=Homo sapiens GN=TRPM6 PE=1 SV=2 | Q9BX84 | 3 | 10 |  |
| Trifunctional enzyme subunit beta, mitochondrial OS=Mus musculus GN=Hadhb PE=1 SV=1 | Q99JY0 | 3 | ON |  |
| Tropomodulin-3 OS=Mus musculus GN=Tmod3 PE=1 SV=1 | Q9JHJ0 | 3 | ON-OFF-10 |  |
| U1 small nuclear ribonucleoprotein 70 kDa OS=Homo sapiens GN=SNRP70 PE=1 SV=2 | P08621 | 3 | ON | OFF-10 |
| Ubiquilin-1 OS=Rattus norvegicus GN=Ubqln1 PE=1 SV=1 | Q9JJP9 | 3 | OFF-10 |  |
| Uncharacterized protein KIAA0082 OS=Mus musculus GN=Kiaa0082 PE=1 SV=1 | Q9DBC3 | 3 |  | ON |
| Uveal autoantigen with coiled-coil domains and ankyrin repeats OS=Homo sapiens GN=UACA PE=1 SV=2 | Q9BZF9 | 3 | ON |  |
| Vacuolar protein sorting-associated protein 13D OS=Homo sapiens GN=VPS13D PE=1 SV=1 | Q5THJ4 | 3 | ON |  |
| Vigilin OS=Rattus norvegicus GN=Hdlbp PE=1 SV=1 | Q9Z1A6 | 3 |  | OFF-10 |
| Xin actin-binding repeat-containing protein 2 OS=Mus musculus GN=Xirp2 PE=1 SV=1 | Q4U4S6 | 3 | OFF |  |
| Zinc finger protein 318 OS=Homo sapiens GN=ZNF318 PE=1 SV=2 | Q5VUA4 | 3 |  | OFF |
| Zinc finger protein 592 OS=Mus musculus GN=Znf592 PE=1 SV=2 | Q8BHZ4 | 3 |  | OFF |
| 1-phosphatidylinositol-4,5-bisphosphate phosphodiesterase beta-1 OS=Homo sapiens GN=PLCB1 PE=1 SV=1 | Q9NQ66 | 2 |  | OFF-10 |
| 14-3-3 protein beta/alpha OS=Homo sapiens GN=YWHAB PE=1 SV=3 | P31946 | 2 | ON-OFF |  |
| 26S proteasome non-ATPase regulatory subunit 2 OS=Homo sapiens GN=PSMD2 PE=1 SV=3 | Q13200 | 2 | ON-OFF-10 |  |
| 3-hydroxy-3-methylglutaryl-coenzyme A reductase OS=Rattus norvegicus GN=Hmgcr PE=1 SV=2 | P51639 | 2 | ON |  |
| 40S ribosomal protein S13 OS=Cricetulus griseus GN=RPS13 PE=3 SV=3 | Q9WVH0 | 2 | ON-OFF-10 | ON-OFF-10 |
| 40S ribosomal protein S17 OS=Canis familiaris GN=RPS17 PE=2 SV=2 | P63273 | 2 | OFF-10 | 10 |
| 40S ribosomal protein S24 OS=Homo sapiens GN=RPS24 PE=1 SV=1 | P62847 | 2 | ON-OFF-10 |  |
| 60S acidic ribosomal protein P1 OS=Homo sapiens GN=RPLP1 PE=1 SV=1 | P05386 | 2 | ON-OFF-10 | 10 |
| 60S ribosomal protein L12 OS=Homo sapiens GN=RPL12 PE=1 SV=1 | P30050 | 2 | ON-OFF-10 | 10 |
| 60S ribosomal protein L13a (Fragment) OS=Sus scrofa GN=RPL13A PE=2 SV=2 | Q95307 | 2 | OFF |  |
| 60S ribosomal protein L14 OS=Sus scrofa GN=RPL14 PE=2 SV=1 | A1XQU3 | 2 | OFF |  |
| 60S ribosomal protein L18 OS=Homo sapiens GN=RPL18 PE=1 SV=2 | Q07020 | 2 |  | OFF-10 |
| 60S ribosomal protein L27 OS=Canis familiaris GN=RPL27 PE=2 SV=3 | Q9XSU7 | 2 | ON-OFF |  |
| 60S ribosomal protein L27a OS=Homo sapiens GN=RPL27A PE=1 SV=2 | P46776 | 2 | ON | OFF-10 |
| 60S ribosomal protein L28 OS=Homo sapiens GN=RPL28 PE=1 SV=3 | P46779 | 2 | ON-OFF-10 | OFF-10 |
| 60S ribosomal protein L31 OS=Homo sapiens GN=RPL31 PE=1 SV=1 | P62899 | 2 | ON | ON-OFF-10 |
| 60S ribosomal protein L35 OS=Sus scrofa GN=RPL35 PE=2 SV=3 | Q29361 | 2 |  | ON-OFF |
| 60S ribosomal protein L36a OS=Homo sapiens GN=RPL36A PE=1 SV=2 | P83881 | 2 |  | ON-OFF |
| 60S ribosomal protein L37a OS=Homo sapiens GN=RPL37A PE=1 SV=2 | P61513 | 2 |  | ON-OFF |
| 60S ribosomal protein L8 OS=Homo sapiens GN=RPL8 PE=1 SV=2 | P62917 | 2 | ON-OFF | ON-OFF |
| A disintegrin and metalloproteinase with thrombospondin motifs 1 OS=Homo sapiens GN=ADAMTS1 PE=1 SV=4 | Q9UHI8 | 2 |  | OFF |
| Activated RNA polymerase II transcriptional coactivator p15 OS=Macaca fascicularis GN=SUB1 PE=2 SV=1 | Q4R947 | 2 | ON-OFF-10 | 10 |
| ADP/ATP translocase 2 OS=Homo sapiens GN=SLC25A5 PE=1 SV=6 | P05141 | 2 | 10 |  |
| Agrin OS=Rattus norvegicus GN=Agrn PE=2 SV=2 | P25304 | 2 |  | 10 |
| Alpha-centractin OS=Homo sapiens GN=ACTR1A PE=1 SV=1 | P61163 | 2 | ON |  |
| Alpha-fetoprotein OS=Equus caballus GN=AFP PE=2 SV=1 | P49066 | 2 | ON | 10 |
| Alpha-internexin OS=Rattus norvegicus GN=Ina PE=1 SV=2 | P23565 | 2 | ON-OFF | 10 |
| Alpha-S1-casein OS=Bubalus bubalis GN=CSN1S1 PE=2 SV=2 | O62823 | 2 | 10 |  |
| ALX homeobox protein 1 OS=Mus musculus GN=Alx1 PE=2 SV=1 | Q8C8B0 | 2 |  | 10 |
| Aminopeptidase N OS=Sus scrofa GN=ANPEP PE=1 SV=3 | P15145 | 2 | OFF |  |
| Astrocytic phosphoprotein PEA-15 OS=Homo sapiens GN=PEA15 PE=1 SV=2 | Q15121 | 2 | ON-OFF |  |
| ATP synthase subunit d, mitochondrial OS=Homo sapiens GN=ATP5H PE=1 SV=3 | O75947 | 2 | OFF |  |
| ATP-binding cassette sub-family A member 2 OS=Mus musculus GN=Abca2 PE=2 SV=4 | P41234 | 2 | OFF |  |
| ATP-binding cassette sub-family B member 7, mitochondrial OS=Rattus norvegicus GN=Abcb7 PE=2 SV=1 | Q704E8 | 2 | OFF |  |
| Barrier-to-autointegration factor OS=Homo sapiens GN=BANF1 PE=1 SV=1 | O75531 | 2 | ON-OFF-10 | ON |
| BAT2 domain-containing protein 1 OS=Homo sapiens GN=BAT2D1 PE=1 SV=2 | Q9Y520 | 2 | OFF-10 |  |
| Brain acid soluble protein 1 OS=Bos taurus GN=BASP1 PE=1 SV=3 | P80724 | 2 | ON-OFF-10 |  |
| Brain-specific angiogenesis inhibitor 1-associated protein 2-like protein 2 OS=Homo sapiens GN=BAIAP2L2 PE=2 SV=1 | Q6UXY1 | 2 |  | OFF |
| Bromodomain adjacent to zinc finger domain protein 1B OS=Mus musculus GN=Baz1b PE=1 SV=1 | Q9Z277 | 2 | OFF |  |
| Cadherin EGF LAG seven-pass G-type receptor 1 OS=Homo sapiens GN=CELSR1 PE=2 SV=1 | Q9NYQ6 | 2 | ON |  |
| Calnexin OS=Mus musculus GN=Canx PE=1 SV=1 | P35564 | 2 | ON-OFF-10 |  |
| Calpastatin OS=Sus scrofa GN=CAST PE=2 SV=1 | P12675 | 2 | OFF-10 |  |
| Calreticulin (Fragment) OS=Sus scrofa GN=CALR PE=1 SV=2 | P28491 | 2 | ON-OFF |  |
| Caprin-1 OS=Homo sapiens GN=CAPRIN1 PE=1 SV=2 | Q14444 | 2 |  | 10 |
| Casein kinase II subunit beta OS=Homo sapiens GN=CSNK2B PE=1 SV=1 | P67870 | 2 | ON |  |
| Cathepsin D OS=Bos taurus GN=CTSD PE=1 SV=2 | P80209 | 2 | ON-OFF-10 |  |
| Cell cycle checkpoint protein RAD17 OS=Cercopithecus aethiops GN=RAD17 PE=1 SV=1 | Q9XT62 | 2 | 10 |  |
| Chloride intracellular channel protein 4 OS=Rattus norvegicus GN=Clic4 PE=2 SV=3 | Q9Z0W7 | 2 | ON |  |
| Chromodomain-helicase-DNA-binding protein 7 OS=Homo sapiens GN=CHD7 PE=1 SV=3 | Q9P2D1 | 2 | ON |  |
| Clathrin light chain B OS=Homo sapiens GN=CLTB PE=1 SV=1 | P09497 | 2 | ON-OFF-10 | OFF-10 |
| Cleavage and polyadenylation specificity factor subunit 5 OS=Homo sapiens GN=NUDT21 PE=1 SV=1 | O43809 | 2 | OFF |  |
| Coiled-coil domain-containing protein 45 OS=Homo sapiens GN=CCDC45 PE=1 SV=1 | Q96GE4 | 2 |  | OFF |
| Coiled-coil domain-containing protein 46 OS=Mus musculus GN=Ccdc46 PE=2 SV=2 | Q5PR68 | 2 | 10 |  |
| Coiled-coil domain-containing protein 52 OS=Bos taurus GN=CCDC52 PE=2 SV=1 | Q2T9X8 | 2 | ON |  |
| Coiled-coil domain-containing protein 94 OS=Homo sapiens GN=CCDC94 PE=1 SV=1 | Q9BW85 | 2 | 10 |  |
| Cold-inducible RNA-binding protein OS=Pongo abelii GN=CIRBP PE=2 SV=1 | Q5RF83 | 2 | OFF-10 | 10 |
| Collagen alpha-1(XVII) chain OS=Canis familiaris GN=COL17A1 PE=2 SV=2 | Q9N281 | 2 |  | 10 |
| Cone-rod homeobox protein OS=Canis familiaris GN=CRX PE=2 SV=1 | Q8SQ03 | 2 |  | OFF |
| Cortactin-binding protein 2 OS=Ateles geoffroyi GN=CTTNBP2 PE=3 SV=1 | Q09YK4 | 2 | 10 | OFF |
| Cystathionine gamma-lyase OS=Sus scrofa GN=CTH PE=2 SV=1 | Q19QT7 | 2 | ON |  |
| Cysteine and glycine-rich protein 1 OS=Bos taurus GN=CSRP1 PE=2 SV=3 | Q3MHY1 | 2 |  | 10 |
| Cytochrome c OS=Sus scrofa GN=CYCS PE=1 SV=2 | P62895 | 2 |  | ON-OFF-10 |
| Cytochrome c oxidase subunit VIb isoform 1 OS=Tarsius syrichta GN=COX6B1 PE=3 SV=3 | Q7YRK6 | 2 | 10 |  |
| Cytochrome P450 2C4 OS=Oryctolagus cuniculus GN=CYP2C4 PE=2 SV=1 | P11371 | 2 | 10 |  |
| Cytochrome P450 2J2 OS=Homo sapiens GN=CYP2J2 PE=1 SV=2 | P51589 | 2 |  | OFF |
| Cytoskeleton-associated protein 4 OS=Homo sapiens GN=CKAP4 PE=1 SV=2 | Q07065 | 2 |  | 10 |
| Cytoskeleton-associated protein 4 OS=Homo sapiens GN=CKAP4 PE=1 SV=2 | Q07065 | 2 | ON-OFF-10 |  |
| Deoxynucleotidyltransferase terminal-interacting protein 2 OS=Homo sapiens GN=DNTTIP2 PE=1 SV=2 | Q5QJE6 | 2 |  | OFF-10 |
| Dihydrolipoyl dehydrogenase, mitochondrial OS=Sus scrofa GN=DLD PE=1 SV=1 | P09623 | 2 | OFF |  |
| Dihydrolipoyllysine-residue acetyltransferase component of pyruvate dehydrogenase complex, mitochondrial OS=Homo sapiens GN=DLAT PE=1 SV=2 | P10515 | 2 | ON-OFF-10 |  |
| Dihydropyrimidinase-related protein 2 OS=Mus musculus GN=Dpysl2 PE=1 SV=2 | O08553 | 2 | OFF-10 |  |
| Dimethyladenosine transferase 1, mitochondrial OS=Rattus norvegicus GN=Tfb1m PE=2 SV=1 | Q811P6 | 2 | OFF |  |
| DIS3-like exonuclease 1 OS=Mus musculus GN=Dis3l PE=2 SV=2 | Q8C0S1 | 2 |  | OFF |
| DNA (cytosine-5)-methyltransferase 3A OS=Rattus norvegicus GN=Dnmt3a PE=2 SV=1 | Q1LZ53 | 2 | ON |  |
| DNA topoisomerase 1 OS=Homo sapiens GN=TOP1 PE=1 SV=2 | P11387 | 2 |  | OFF-10 |
| DNA-directed RNA polymerase II subunit RPB9 OS=Mus musculus GN=Polr2i PE=2 SV=1 | P60898 | 2 |  | 10 |
| DnaJ homolog subfamily C member 8 OS=Mus musculus GN=Dnajc8 PE=2 SV=2 | Q6NZB0 | 2 | OFF |  |
| Drebrin OS=Rattus norvegicus GN=Dbn1 PE=2 SV=3 | Q07266 | 2 | 10 |  |
| Dynein heavy chain 1, axonemal OS=Rattus norvegicus GN=Dnah1 PE=2 SV=2 | Q63164 | 2 | 10 |  |
| Dynein heavy chain 10, axonemal OS=Homo sapiens GN=DNAH10 PE=2 SV=3 | Q8IVF4 | 2 |  | OFF |
| Dynein heavy chain 11, axonemal OS=Homo sapiens GN=DNAH11 PE=1 SV=2 | Q96DT5 | 2 | OFF |  |
| ELAV-like protein 1 OS=Homo sapiens GN=ELAVL1 PE=1 SV=2 | Q15717 | 2 | ON-OFF | OFF |
| Elongation factor 1-delta OS=Ovis aries GN=EEF1D PE=2 SV=1 | Q717R8 | 2 | ON-OFF-10 | 10 |
| Elongation factor Tu, mitochondrial OS=Bos taurus GN=TUFM PE=1 SV=1 | P49410 | 2 | ON-OFF |  |
| Eukaryotic translation initiation factor 3 subunit C OS=Homo sapiens GN=EIF3C PE=1 SV=1 | Q99613 | 2 | ON-OFF-10 | ON-OFF |
| Eukaryotic translation initiation factor 3 subunit I OS=Homo sapiens GN=EIF3I PE=1 SV=1 | Q13347 | 2 | ON-OFF |  |
| Eukaryotic translation initiation factor 4 gamma 3 OS=Homo sapiens GN=EIF4G3 PE=1 SV=2 | O43432 | 2 | 10 |  |
| F-actin-capping protein subunit beta OS=Mus musculus GN=Capzb PE=1 SV=3 | P47757 | 2 | ON-OFF-10 | 10 |
| FAM75-like protein C9orf79 OS=Homo sapiens GN=C9orf79 PE=2 SV=2 | Q6ZUB1 | 2 |  | 10 |
| Far upstream element-binding protein 2 OS=Mus musculus GN=Khsrp PE=1 SV=1 | Q3U0V1 | 2 | 10 | 10 |
| Fer-1-like protein 4 OS=Mus musculus GN=Fer1l4 PE=2 SV=2 | A3KGK3 | 2 | ON |  |
| Ferritin light chain (Fragment) OS=Sus scrofa GN=FTL PE=1 SV=1 | P19133 | 2 | OFF |  |
| Filensin OS=Homo sapiens GN=BFSP1 PE=1 SV=3 | Q12934 | 2 | OFF |  |
| FYVE, RhoGEF and PH domain-containing protein 1 OS=Mus musculus GN=Fgd1 PE=1 SV=1 | P52734 | 2 | OFF |  |
| Gem-associated protein 5 OS=Homo sapiens GN=GEMIN5 PE=1 SV=2 | Q8TEQ6 | 2 | OFF-10 |  |
| Glutamate [NMDA] receptor subunit 3A OS=Homo sapiens GN=GRIN3A PE=1 SV=1 | Q8TCU5 | 2 |  | 10 |
| Glutamine and serine-rich protein 1 OS=Homo sapiens GN=QSER1 PE=1 SV=2 | Q2KHR3 | 2 |  | OFF |
| Golgin subfamily A member 4 OS=Homo sapiens GN=GOLGA4 PE=1 SV=1 | Q13439 | 2 |  | OFF |
| Heat shock 70 kDa protein 4L OS=Mus musculus GN=Hspa4l PE=1 SV=2 | P48722 | 2 | ON |  |
| Heat shock protein beta-6 OS=Homo sapiens GN=HSPB6 PE=1 SV=2 | O14558 | 2 | OFF-10 |  |
| Heat shock protein HSP 90-alpha (Fragment) OS=Equus caballus GN=HSP90AA1 PE=2 SV=1 | Q9GKX7 | 2 | ON |  |
| Heterogeneous nuclear ribonucleoprotein H3 OS=Homo sapiens GN=HNRNPH3 PE=1 SV=2 | P31942 | 2 | ON-OFF-10 |  |
| Heterogeneous nuclear ribonucleoprotein L OS=Mus musculus GN=Hnrnpl PE=1 SV=1 | Q8R081 | 2 | ON |  |
| Heterogeneous nuclear ribonucleoprotein U-like protein 2 OS=Mus musculus GN=Hnrnpul2 PE=1 SV=1 | Q00PI9 | 2 |  | OFF-10 |
| Heterogeneous nuclear ribonucleoproteins C1/C2 OS=Mus musculus GN=Hnrnpc PE=1 SV=1 | Q9Z204 | 2 |  | 10 |
| Histone H1.2 OS=Rattus norvegicus GN=Hist1h1c PE=1 SV=3 | P15865 | 2 |  | ON |
| Histone H1t OS=Rattus norvegicus GN=Hist1h1t PE=1 SV=2 | P06349 | 2 |  | 10 |
| Histone H1x OS=Homo sapiens GN=H1FX PE=1 SV=1 | Q92522 | 2 | ON |  |
| Inositol monophosphatase OS=Sus scrofa GN=IMPA1 PE=2 SV=1 | O77591 | 2 | ON-OFF |  |
| Intersectin-2 OS=Mus musculus GN=Itsn2 PE=1 SV=1 | Q9Z0R6 | 2 |  | OFF |
| Keratin, type I cytoskeletal 25 OS=Pan troglodytes GN=krt25a PE=2 SV=1 | A5A6N2 | 2 | 10 |  |
| Keratin, type II cuticular Hb2 OS=Mus musculus GN=Krt82 PE=2 SV=1 | Q99M74 | 2 | OFF |  |
| Keratin, type II cuticular Hb5 OS=Mus musculus GN=Krt85 PE=2 SV=2 | Q9Z2T6 | 2 | OFF |  |
| Keratin, type II cytoskeletal 3 OS=Homo sapiens GN=KRT3 PE=1 SV=2 | P12035 | 2 | 10 | 10 |
| Keratin, type II cytoskeletal 71 OS=Mus musculus GN=Krt71 PE=1 SV=1 | Q9R0H5 | 2 |  | OFF |
| Keratin, type II cytoskeletal 72 OS=Bos taurus GN=KRT72 PE=2 SV=1 | Q148H8 | 2 | ON |  |
| Keratin, type II cytoskeletal 73 OS=Mus musculus GN=Krt73 PE=2 SV=1 | Q6NXH9 | 2 | 10 |  |
| Keratin, type II cytoskeletal 74 OS=Homo sapiens GN=KRT74 PE=1 SV=2 | Q7RTS7 | 2 | OFF-10 |  |
| Keratin, type II cytoskeletal 8 (Fragment) OS=Potorous tridactylus GN=KRT8 PE=2 SV=1 | Q28810 | 2 |  | OFF |
| Kinesin-like protein KIF1B OS=Mus musculus GN=Kif1b PE=2 SV=2 | Q60575 | 2 |  | 10 |
| Kinesin-like protein KIF21A OS=Homo sapiens GN=KIF21A PE=1 SV=2 | Q7Z4S6 | 2 | ON |  |
| Kinesin-like protein KIF22 OS=Bos taurus GN=KIF22 PE=2 SV=2 | A6QPL4 | 2 | OFF |  |
| Lactase-phlorizin hydrolase OS=Rattus norvegicus GN=Lct PE=2 SV=2 | Q02401 | 2 | 10 |  |
| Laminin subunit alpha-1 OS=Homo sapiens GN=LAMA1 PE=1 SV=1 | P25391 | 2 | OFF |  |
| Large proline-rich protein BAT2 OS=Rattus norvegicus GN=Bat2 PE=1 SV=1 | Q6MG48 | 2 | ON |  |
| Lysosomal alpha-mannosidase OS=Bos taurus GN=MAN2B1 PE=1 SV=3 | Q29451 | 2 | OFF |  |
| Malate dehydrogenase, cytoplasmic OS=Felis silvestris catus GN=MDH1 PE=2 SV=3 | Q7YRU4 | 2 | ON |  |
| MAM domain-containing glycosylphosphatidylinositol anchor protein 2 OS=Homo sapiens GN=MDGA2 PE=2 SV=2 | Q7Z553 | 2 | ON-10 |  |
| Membrane-spanning 4-domains subfamily A member 14 OS=Homo sapiens GN=MS4A14 PE=2 SV=1 | Q96JA4 | 2 | OFF |  |
| MICAL C-terminal-like protein OS=Homo sapiens GN=MICALCL PE=2 SV=2 | Q6ZW33 | 2 | ON |  |
| Microtubule-associated protein 2 OS=Mus musculus GN=Map2 PE=1 SV=1 | P20357 | 2 | OFF-10 | 10 |
| Microtubule-associated protein RP/EB family member 1 OS=Bos taurus GN=MAPRE1 PE=2 SV=3 | Q3ZBD9 | 2 | ON-OFF |  |
| Mitochondrial import inner membrane translocase subunit Tim8 A OS=Homo sapiens GN=TIMM8A PE=1 SV=1 | O60220 | 2 | OFF-10 |  |
| Myoferlin OS=Mus musculus GN=Fer1l3 PE=2 SV=2 | Q69ZN7 | 2 | ON-OFF-10 |  |
| Myosin light chain 1, skeletal muscle isoform OS=Mus musculus GN=Myl1 PE=1 SV=2 | P05977 | 2 |  | 10 |
| Myosin light chain 3 OS=Rattus norvegicus GN=Myl3 PE=1 SV=2 | P16409 | 2 | ON-OFF-10 | OFF-10 |
| Myosin phosphatase Rho-interacting protein OS=Homo sapiens GN=MPRIP PE=1 SV=2 | Q6WCQ1 | 2 | 10 |  |
| Myosin-14 OS=Homo sapiens GN=MYH14 PE=1 SV=1 | Q7Z406 | 2 | OFF | OFF |
| NADH dehydrogenase [ubiquinone] 1 alpha subcomplex subunit 8 OS=Homo sapiens GN=NDUFA8 PE=1 SV=3 | P51970 | 2 | ON-OFF-10 |  |
| Nascent polypeptide-associated complex subunit alpha OS=Chinchilla lanigera PE=2 SV=1 | Q6QN10 | 2 | ON-OFF-10 | OFF-10 |
| Neurofilament medium polypeptide OS=Bos taurus GN=NEFM PE=1 SV=3 | O77788 | 2 | ON |  |
| Nexilin OS=Homo sapiens GN=NEXN PE=1 SV=1 | Q0ZGT2 | 2 | OFF | 10 |
| NFX1-type zinc finger-containing protein 1 OS=Mus musculus GN=Znfx1 PE=2 SV=2 | Q8R151 | 2 | ON |  |
| Nipped-B-like protein OS=Homo sapiens GN=NIPBL PE=1 SV=2 | Q6KC79 | 2 | OFF |  |
| Nuclear mitotic apparatus protein 1 OS=Homo sapiens GN=NUMA1 PE=1 SV=2 | Q14980 | 2 | OFF |  |
| Nucleobindin-1 OS=Mus musculus GN=Nucb1 PE=1 SV=2 | Q02819 | 2 | OFF-10 |  |
| Nucleolar protein 5 OS=Macaca fascicularis GN=NOL5 PE=2 SV=1 | Q4R779 | 2 | 10 |  |
| Nucleolar RNA helicase 2 OS=Homo sapiens GN=DDX21 PE=1 SV=5 | Q9NR30 | 2 | ON | 10 |
| Nucleolysin TIA-1 OS=Mus musculus GN=Tia1 PE=1 SV=1 | P52912 | 2 | ON |  |
| Nucleoprotein OS=Human SARS coronavirus GN=N PE=1 SV=1 | P59595 | 2 |  | ON |
| Nucleoprotein TPR OS=Homo sapiens GN=TPR PE=1 SV=2 | P12270 | 2 | ON |  |
| Nucleoside diphosphate kinase A OS=Mus musculus GN=Nme1 PE=1 SV=1 | P15532 | 2 | ON-OFF |  |
| Nucleosome assembly protein 1-like 1 OS=Homo sapiens GN=NAP1L1 PE=1 SV=1 | P55209 | 2 | OFF |  |
| Oral-facial-digital syndrome 1 protein homolog OS=Mus musculus GN=Ofd1 PE=2 SV=1 | Q80Z25 | 2 | OFF |  |
| Origin recognition complex subunit 1 OS=Cricetulus griseus GN=ORC1L PE=2 SV=1 | Q9JI69 | 2 |  | 10 |
| Peroxiredoxin-4 OS=Homo sapiens GN=PRDX4 PE=1 SV=1 | Q13162 | 2 | ON-OFF |  |
| Phosphoglycerate mutase 2 OS=Homo sapiens GN=PGAM2 PE=1 SV=3 | P15259 | 2 | 10 |  |
| Phosphoserine phosphatase OS=Mus musculus GN=Psph PE=2 SV=1 | Q99LS3 | 2 | ON |  |
| Pleckstrin homology domain-containing family G member 1 OS=Homo sapiens GN=PLEKHG1 PE=1 SV=2 | Q9ULL1 | 2 | OFF |  |
| Polyamine-modulated factor 1-binding protein 1 OS=Homo sapiens GN=PMFBP1 PE=2 SV=1 | Q8TBY8 | 2 | 10 |  |
| Polypyrimidine tract-binding protein 1 OS=Sus scrofa GN=PTBP1 PE=2 SV=1 | Q29099 | 2 | ON-10 | 10 |
| PRA1 family protein 3 OS=Sus scrofa GN=ARL6IP5 PE=2 SV=1 | Q56P28 | 2 | 10 |  |
| Proactivator polypeptide OS=Homo sapiens GN=PSAP PE=1 SV=2 | P07602 | 2 | OFF-10 |  |
| Probable global transcription activator SNF2L1 OS=Mus musculus GN=Smarca1 PE=1 SV=1 | Q6PGB8 | 2 | OFF |  |
| Probable glutamyl-tRNA synthetase, mitochondrial OS=Homo sapiens GN=EARS2 PE=2 SV=2 | Q5JPH6 | 2 | 10 |  |
| Probable Ras GTPase-activating protein FLJ21438 OS=Homo sapiens PE=2 SV=2 | Q86YV0 | 2 |  | 10 |
| Proteasome subunit alpha type-5 OS=Homo sapiens GN=PSMA5 PE=1 SV=3 | P28066 | 2 | ON |  |
| Proteasome subunit alpha type-7 OS=Homo sapiens GN=PSMA7 PE=1 SV=1 | O14818 | 2 | ON |  |
| Proteasome subunit beta type-4 OS=Bos taurus GN=PSMB4 PE=1 SV=1 | Q3T108 | 2 | ON |  |
| Protein BTG3 OS=Sus scrofa GN=BTG3 PE=2 SV=1 | A4UTQ2 | 2 |  | ON |
| Protein C15orf2 OS=Homo sapiens GN=C15orf2 PE=2 SV=2 | Q9NZP6 | 2 | 10 |  |
| Protein CASC5 OS=Homo sapiens GN=CASC5 PE=1 SV=2 | Q8NG31 | 2 | ON |  |
| Protein kinase C iota type OS=Mus musculus GN=Prkci PE=1 SV=2 | Q62074 | 2 | 10 |  |
| Protein LAP4 OS=Mus musculus GN=Scrib PE=1 SV=2 | Q80U72 | 2 | OFF |  |
| Protein S-myc OS=Mus musculus GN=Mycs PE=2 SV=1 | Q9Z304 | 2 | ON |  |
| Protein S100-A2 OS=Bos taurus GN=S100A2 PE=1 SV=1 | P10462 | 2 | ON-OFF-10 |  |
| Protein transport protein Sec23A OS=Homo sapiens GN=SEC23A PE=1 SV=2 | Q15436 | 2 | ON-OFF |  |
| Prothymosin alpha OS=Homo sapiens GN=PTMA PE=1 SV=2 | P06454 | 2 | ON-OFF-10 |  |
| Proto-oncogene C-crk OS=Rattus norvegicus GN=Crk PE=1 SV=1 | Q63768 | 2 | 10 |  |
| Protocadherin 18 OS=Mus musculus GN=Pcdh18 PE=1 SV=2 | Q8VHR0 | 2 |  | 10 |
| Putative RNA-binding protein 3 OS=Mus musculus GN=Rbm3 PE=2 SV=1 | O89086 | 2 | OFF |  |
| Putative tubulin-like protein alpha-4B OS=Homo sapiens GN=TUBA4B PE=5 SV=2 | Q9H853 | 2 | ON-OFF |  |
| Rab proteins geranylgeranyltransferase component A 1 OS=Homo sapiens GN=CHM PE=1 SV=3 | P24386 | 2 |  | OFF |
| Ras-related protein Ral-B OS=Macaca fascicularis GN=RALB PE=2 SV=1 | Q4R379 | 2 | 10 |  |
| RAS-responsive element-binding protein 1 OS=Homo sapiens GN=RREB1 PE=1 SV=3 | Q92766 | 2 | ON |  |
| Receptor-type tyrosine-protein phosphatase beta OS=Homo sapiens GN=PTPRB PE=1 SV=1 | P23467 | 2 | 10 |  |
| Reticulocalbin-1 OS=Homo sapiens GN=RCN1 PE=1 SV=1 | Q15293 | 2 | ON-10 |  |
| Reticulon-4 OS=Homo sapiens GN=RTN4 PE=1 SV=2 | Q9NQC3 | 2 | 10 |  |
| Retinoblastoma-like protein 2 OS=Rattus norvegicus GN=Rbl2 PE=1 SV=1 | O55081 | 2 | OFF-10 |  |
| Rho GTPase-activating protein 21 OS=Mus musculus GN=Arhgap21 PE=1 SV=1 | Q6DFV3 | 2 | 10 |  |
| Rho guanine nucleotide exchange factor 11 OS=Rattus norvegicus GN=Arhgef11 PE=1 SV=1 | Q9ES67 | 2 |  | OFF |
| Rho guanine nucleotide exchange factor 19 OS=Homo sapiens GN=ARHGEF19 PE=2 SV=1 | Q8IW93 | 2 | OFF |  |
| Rhodopsin OS=Macaca fascicularis GN=RHO PE=2 SV=1 | Q28886 | 2 | OFF |  |
| Ribonucleoside-diphosphate reductase large subunit OS=Homo sapiens GN=RRM1 PE=1 SV=1 | P23921 | 2 | ON |  |
| Ribosome biogenesis regulatory protein homolog OS=Homo sapiens GN=RRS1 PE=1 SV=2 | Q15050 | 2 | 10 |  |
| RING finger protein 168 OS=Mus musculus GN=Rnf168 PE=2 SV=2 | Q80XJ2 | 2 | ON |  |
| RNA-binding protein 39 OS=Pongo abelii GN=RBM39 PE=2 SV=1 | Q5RC80 | 2 | ON-OFF | 10 |
| RNA-binding protein NOB1 OS=Rattus norvegicus GN=Nob1 PE=2 SV=1 | Q6VEU1 | 2 |  | ON |
| RNA-binding protein Raly OS=Mus musculus GN=Raly PE=1 SV=2 | Q64012 | 2 |  | OFF-10 |
| RNA-binding protein with serine-rich domain 1 OS=Macaca fascicularis GN=RNPS1 PE=2 SV=1 | Q4R5N1 | 2 |  | OFF-10 |
| Sacsin OS=Homo sapiens GN=SACS PE=1 SV=2 | Q9NZJ4 | 2 |  | 10 |
| Saposin-B-Val OS=Sus scrofa GN=PSAP PE=1 SV=2 | P81405 | 2 | ON-OFF-10 |  |
| Septin-9 OS=Homo sapiens GN=SEPT9 PE=1 SV=2 | Q9UHD8 | 2 | ON-OFF-10 |  |
| SH3 and multiple ankyrin repeat domains protein 1 OS=Rattus norvegicus GN=Shank1 PE=1 SV=1 | Q9WV48 | 2 |  | 10 |
| Slit homolog 1 protein OS=Homo sapiens GN=SLIT1 PE=2 SV=4 | O75093 | 2 | ON |  |
| Small nuclear ribonucleoprotein Sm D1 OS=Homo sapiens GN=SNRPD1 PE=1 SV=1 | P62314 | 2 | ON-OFF |  |
| SNW domain-containing protein 1 OS=Homo sapiens GN=SNW1 PE=1 SV=1 | Q13573 | 2 | OFF-10 |  |
| Spermatid perinuclear RNA-binding protein OS=Homo sapiens GN=STRBP PE=1 SV=1 | Q96SI9 | 2 |  | OFF |
| Splicing factor 3B subunit 1 OS=Homo sapiens GN=SF3B1 PE=1 SV=2 | O75533 | 2 | 10 | 10 |
| Splicing factor U2AF 65 kDa subunit OS=Mus musculus GN=U2af2 PE=1 SV=3 | P26369 | 2 | ON-OFF-10 | ON-OFF-10 |
| Stathmin OS=Homo sapiens GN=STMN1 PE=1 SV=3 | P16949 | 2 | ON |  |
| STE20-like serine/threonine-protein kinase OS=Rattus norvegicus GN=Slk PE=1 SV=1 | O08815 | 2 | ON |  |
| Stress-induced-phosphoprotein 1 OS=Cricetulus griseus GN=STIP1 PE=2 SV=1 | O54981 | 2 | ON-OFF |  |
| Stromal cell-derived factor 2 OS=Homo sapiens GN=SDF2 PE=2 SV=2 | Q99470 | 2 | ON-OFF |  |
| Structural maintenance of chromosomes flexible hinge domain-containing protein 1 OS=Homo sapiens GN=SMCHD1 PE=1 SV=2 | A6NHR9 | 2 |  | 10 |
| Succinate dehydrogenase [ubiquinone] flavoprotein subunit, mitochondrial OS=Homo sapiens GN=SDHA PE=1 SV=2 | P31040 | 2 | ON |  |
| Synaptonemal complex protein 1 OS=Rattus norvegicus GN=Sycp1 PE=2 SV=2 | Q03410 | 2 | OFF |  |
| T-complex protein 1 subunit alpha B OS=Mus musculus GN=Cct1 PE=1 SV=3 | P11983 | 2 | OFF |  |
| Testican-3 OS=Mus musculus GN=Spock3 PE=2 SV=2 | Q8BKV0 | 2 | 10 |  |
| Tetratricopeptide repeat protein 24 OS=Homo sapiens GN=TTC24 PE=2 SV=1 | A2A3L6 | 2 | OFF |  |
| Tetratricopeptide repeat protein 5 OS=Bos taurus GN=TTC5 PE=2 SV=1 | Q0P5H9 | 2 | 10 |  |
| Thioredoxin domain-containing protein 4 OS=Mus musculus GN=Txndc4 PE=1 SV=1 | Q9D1Q6 | 2 | ON-OFF |  |
| Thioredoxin domain-containing protein 5 OS=Homo sapiens GN=TXNDC5 PE=1 SV=2 | Q8NBS9 | 2 | ON-OFF-10 |  |
| Thioredoxin reductase 1, cytoplasmic OS=Sus scrofa GN=TXNRD1 PE=2 SV=3 | Q9MYY8 | 2 | ON |  |
| Thioredoxin, mitochondrial OS=Mus musculus GN=Txn2 PE=2 SV=1 | P97493 | 2 | ON |  |
| Thyroid receptor-interacting protein 11 OS=Homo sapiens GN=TRIP11 PE=1 SV=2 | Q15643 | 2 | OFF |  |
| Toll-interacting protein OS=Homo sapiens GN=TOLLIP PE=1 SV=1 | Q9H0E2 | 2 | ON-OFF |  |
| Trafficking protein particle complex subunit 10 OS=Mus musculus GN=Trappc10 PE=2 SV=2 | Q3TLI0 | 2 | 10 |  |
| Transcription elongation factor B polypeptide 2 OS=Mus musculus GN=Tceb2 PE=1 SV=1 | P62869 | 2 | ON-OFF |  |
| Translationally-controlled tumor protein OS=Homo sapiens GN=TPT1 PE=1 SV=1 | P13693 | 2 | ON-OFF |  |
| Transmembrane channel-like protein 4 OS=Mus musculus GN=Tmc4 PE=2 SV=2 | Q7TQ65 | 2 | ON |  |
| Treacle protein OS=Mus musculus GN=Tcof1 PE=1 SV=1 | O08784 | 2 |  | 10 |
| Tubulin alpha-1A chain OS=Homo sapiens GN=TUBA1A PE=1 SV=1 | Q71U36 | 2 |  | 10 |
| Tubulin alpha-1C chain OS=Homo sapiens GN=TUBA1C PE=1 SV=1 | Q9BQE3 | 2 | 10 |  |
| Tubulin beta-2C chain OS=Homo sapiens GN=TUBB2C PE=1 SV=1 | P68371 | 2 |  | 10 |
| Tumor necrosis factor, alpha-induced protein 8-like protein 1 OS=Bos taurus GN=TNFAIP8L1 PE=2 SV=1 | A5PK29 | 2 | OFF |  |
| U1 small nuclear ribonucleoprotein A OS=Homo sapiens GN=SNRPA PE=1 SV=3 | P09012 | 2 | ON-OFF-10 |  |
| U2 small nuclear ribonucleoprotein A' OS=Macaca fascicularis GN=SNRPA1 PE=2 SV=1 | Q4R8Y8 | 2 | ON-OFF-10 |  |
| U3 small nucleolar ribonucleoprotein protein IMP4 OS=Mus musculus GN=Imp4 PE=2 SV=1 | Q8VHZ7 | 2 |  | OFF |
| UBX domain-containing protein 1 OS=Mus musculus GN=Ubxn1 PE=1 SV=1 | Q922Y1 | 2 | ON-OFF-10 |  |
| UMP-CMP kinase OS=Sus scrofa GN=CMPK1 PE=1 SV=1 | Q29561 | 2 | ON-OFF |  |
| Uncharacterized protein C14orf37 OS=Homo sapiens GN=C14orf37 PE=2 SV=1 | Q86TY3 | 2 | 10 |  |
| Uncharacterized protein KIAA1107 OS=Mus musculus GN=Kiaa1107 PE=2 SV=4 | Q80TK0 | 2 | 10 |  |
| UPF0027 protein C22orf28 OS=Homo sapiens GN=C22orf28 PE=1 SV=1 | Q9Y3I0 | 2 | ON |  |
| UPF0568 protein C14orf166 OS=Homo sapiens GN=C14orf166 PE=1 SV=1 | Q9Y224 | 2 | ON |  |
| UPF0568 protein C14orf166 OS=Homo sapiens GN=C14orf166 PE=1 SV=1 | Q9Y224 | 2 | ON-OFF |  |
| Urotensin-2 OS=Homo sapiens GN=UTS2 PE=1 SV=1 | O95399 | 2 | ON-OFF |  |
| UTP--glucose-1-phosphate uridylyltransferase OS=Cricetulus griseus GN=UGP2 PE=2 SV=3 | O35156 | 2 | 10 |  |
| V-type proton ATPase subunit G 1 OS=Homo sapiens GN=ATP6V1G1 PE=1 SV=3 | O75348 | 2 |  | OFF |
| Vascular endothelial growth factor receptor 2 OS=Mus musculus GN=Kdr PE=1 SV=1 | P35918 | 2 | ON |  |
| WD repeat-containing protein 32 OS=Mus musculus GN=Wdr32 PE=2 SV=1 | A2AKB9 | 2 | 10 |  |
| Zinc finger and BTB domain-containing protein 4 OS=Homo sapiens GN=ZBTB4 PE=1 SV=3 | Q9P1Z0 | 2 | OFF |  |
| Zinc finger and SCAN domain-containing protein 20 OS=Homo sapiens GN=ZSCAN20 PE=2 SV=2 | P17040 | 2 |  | OFF |
| Zinc finger E-box-binding homeobox 2 OS=Mus musculus GN=Zeb2 PE=2 SV=1 | Q9R0G7 | 2 |  | 10 |
| Zinc finger protein 214 OS=Homo sapiens GN=ZNF214 PE=2 SV=1 | Q9UL59 | 2 |  | ON |
| Zinc finger protein 280D OS=Homo sapiens GN=ZNF280D PE=1 SV=2 | Q6N043 | 2 |  | OFF |
| Zinc finger protein 784 OS=Homo sapiens GN=ZNF784 PE=2 SV=1 | Q8NCA9 | 2 | ON-OFF |  |
| Zinc finger RNA-binding protein OS=Mus musculus GN=Zfr PE=1 SV=2 | O88532 | 2 | OFF |  |
| Zyxin OS=Mus musculus GN=Zyx PE=1 SV=1 | Q62523 | 2 | ON-OFF |  |
| 2-oxoglutarate dehydrogenase E1 component, mitochondrial OS=Homo sapiens GN=OGDH PE=1 SV=3 | Q02218 | 1 | ON-OFF-10 |  |
| 40S ribosomal protein S12 OS=Bos taurus GN=RPS12 PE=2 SV=1 | Q76I81 | 1 | ON-OFF-10 | ON-OFF-10 |
| 40S ribosomal protein S14 OS=Homo sapiens GN=RPS14 PE=1 SV=3 | P62263 | 1 |  | ON |
| 40S ribosomal protein S23 OS=Chinchilla lanigera GN=RPS23 PE=2 SV=1 | P62298 | 1 | ON | OFF |
| 40S ribosomal protein S25 OS=Homo sapiens GN=RPS25 PE=1 SV=1 | P62851 | 1 | ON-OFF |  |
| 40S ribosomal protein S27a OS=Cavia porcellus GN=RPS27A PE=3 SV=1 | P62978 | 1 | ON-OFF | ON |
| 40S ribosomal protein S28 OS=Homo sapiens GN=RPS28 PE=1 SV=1 | P62857 | 1 |  | 10 |
| 40S ribosomal protein S5 OS=Homo sapiens GN=RPS5 PE=1 SV=4 | P46782 | 1 |  | 10 |
| 40S ribosomal protein S7 OS=Felis silvestris catus GN=RPS7 PE=2 SV=1 | Q5RT64 | 1 | 10 |  |
| 5'-3' exoribonuclease 2 OS=Pongo abelii GN=XRN2 PE=2 SV=1 | Q5R4L5 | 1 | ON |  |
| 6-phosphofructo-2-kinase/fructose-2,6-biphosphatase 1 OS=Bos taurus GN=PFKFB1 PE=2 SV=1 | P49872 | 1 | ON |  |
| 6-phosphogluconate dehydrogenase, decarboxylating (Fragment) OS=Sus scrofa GN=PGD PE=2 SV=3 | P14332 | 1 | ON-OFF |  |
| 60S acidic ribosomal protein P2 OS=Homo sapiens GN=RPLP2 PE=1 SV=1 | P05387 | 1 | ON-OFF-10 |  |
| 60S ribosomal protein L24 OS=Felis silvestris catus GN=RPL24 PE=2 SV=1 | Q66WF5 | 1 | ON-OFF-10 | ON-OFF-10 |
| 60S ribosomal protein L29 OS=Sus scrofa GN=RPL29 PE=2 SV=4 | Q95281 | 1 |  | ON |
| 60S ribosomal protein L30 OS=Homo sapiens GN=RPL30 PE=1 SV=2 | P62888 | 1 | ON |  |
| 60S ribosomal protein L36 OS=Homo sapiens GN=RPL36 PE=1 SV=3 | Q9Y3U8 | 1 | ON-OFF |  |
| 60S ribosomal protein L38 OS=Homo sapiens GN=RPL38 PE=1 SV=2 | P63173 | 1 |  | ON |
| 60S ribosomal protein L40 OS=Canis familiaris GN=UBA52 PE=3 SV=1 | P63050 | 1 | ON-OFF-10 |  |
| Actin-related protein 2/3 complex subunit 1B OS=Mus musculus GN=Arpc1b PE=2 SV=3 | Q9WV32 | 1 | ON |  |
| Actin-related protein 3 OS=Homo sapiens GN=ACTR3 PE=1 SV=3 | P61158 | 1 | ON |  |
| Alpha-parvin OS=Rattus norvegicus GN=Parva PE=1 SV=2 | Q9HB97 | 1 | OFF |  |
| Annexin A6 OS=Rattus norvegicus GN=Anxa6 PE=1 SV=2 | P48037 | 1 | ON |  |
| ATP synthase protein 8 OS=Sus scrofa GN=MT-ATP8 PE=3 SV=2 | Q35914 | 1 | 10 |  |
| ATP synthase subunit f, mitochondrial OS=Pongo abelii GN=ATP5J2 PE=3 SV=3 | Q5R6T5 | 1 | ON-OFF-10 |  |
| ATP synthase subunit O, mitochondrial OS=Sus scrofa GN=ATP5O PE=1 SV=1 | Q2EN81 | 1 | ON-OFF-10 |  |
| ATP-dependent RNA helicase DDX1 OS=Macaca fascicularis GN=DDX1 PE=2 SV=1 | Q4R7L5 | 1 |  | 10 |
| ATP-dependent RNA helicase DDX3X OS=Homo sapiens GN=DDX3X PE=1 SV=3 | O00571 | 1 | ON |  |
| Autism susceptibility gene 2 protein OS=Homo sapiens GN=AUTS2 PE=2 SV=1 | Q8WXX7 | 1 | OFF |  |
| Beta-glucuronidase OS=Felis silvestris catus GN=GUSB PE=1 SV=1 | O97524 | 1 | OFF |  |
| Bifunctional 3'-phosphoadenosine 5'-phosphosulfate synthetase 1 OS=Homo sapiens GN=PAPSS1 PE=1 SV=2 | O43252 | 1 | ON |  |
| Bifunctional aminoacyl-tRNA synthetase OS=Homo sapiens GN=EPRS PE=1 SV=3 | P07814 | 1 | 10 | 10 |
| Bridging integrator 3 OS=Rattus norvegicus GN=Bin3 PE=2 SV=1 | Q68FW8 | 1 |  | 10 |
| Calpain small subunit 1 OS=Homo sapiens GN=CAPNS1 PE=1 SV=1 | P04632 | 1 | ON-OFF |  |
| Cancer susceptibility candidate protein 1 homolog OS=Macaca fascicularis GN=CASC1 PE=2 SV=1 | Q4R796 | 1 | ON-OFF-10 | ON-10 |
| Catenin delta-1 OS=Mus musculus GN=Ctnnd1 PE=1 SV=2 | P30999 | 1 | OFF |  |
| Cathepsin B OS=Homo sapiens GN=CTSB PE=1 SV=3 | P07858 | 1 | OFF-10 |  |
| Caveolin-1 OS=Bos taurus GN=CAV1 PE=2 SV=1 | P79132 | 1 | OFF-10 |  |
| Centrosomal protein of 170 kDa OS=Homo sapiens GN=CEP170 PE=1 SV=1 | Q5SW79 | 1 | 10 | OFF |
| Charged multivesicular body protein 4b OS=Mus musculus GN=Chmp4b PE=2 SV=2 | Q9D8B3 | 1 |  | 10 |
| Chromobox protein homolog 3 OS=Homo sapiens GN=CBX3 PE=1 SV=4 | Q13185 | 1 | ON |  |
| Clathrin light chain A OS=Mus musculus GN=Clta PE=2 SV=1 | O08585 | 1 | ON-10 | 10 |
| Coiled-coil domain-containing protein 124 OS=Pongo abelii GN=CCDC124 PE=2 SV=1 | Q5R8X8 | 1 |  | OFF-10 |
| Collagen alpha-1(II) chain OS=Homo sapiens GN=COL2A1 PE=1 SV=3 | P02458 | 1 |  | 10 |
| Collagen alpha-3(VI) chain OS=Homo sapiens GN=COL6A3 PE=1 SV=3 | P12111 | 1 | OFF |  |
| COP9 signalosome complex subunit 4 OS=Homo sapiens GN=COPS4 PE=1 SV=1 | Q9BT78 | 1 | ON |  |
| Coronin-1B OS=Pongo abelii GN=CORO1B PE=2 SV=2 | Q5NVK4 | 1 | ON |  |
| Cytochrome b-c1 complex subunit 6, mitochondrial OS=Rattus norvegicus GN=Uqcrh PE=2 SV=1 | Q5M9I5 | 1 | ON-OFF-10 |  |
| Cytochrome c oxidase subunit 5A, mitochondrial OS=Macaca mulatta GN=COX5A PE=2 SV=1 | Q53CF8 | 1 | ON-OFF |  |
| Cytosol aminopeptidase OS=Mus musculus GN=Lap3 PE=1 SV=3 | Q9CPY7 | 1 | OFF |  |
| Dihydropteridine reductase OS=Sus scrofa GN=QDPR PE=2 SV=1 | Q8MJ30 | 1 | OFF |  |
| DNA damage-binding protein 1 OS=Cercopithecus aethiops GN=DDB1 PE=1 SV=1 | P33194 | 1 | ON |  |
| DNA-directed RNA polymerases I, II, and III subunit RPABC3 OS=Mus musculus GN=Polr2h PE=2 SV=3 | Q923G2 | 1 | ON-OFF |  |
| DnaJ homolog subfamily B member 11 OS=Homo sapiens GN=DNAJB11 PE=1 SV=1 | Q9UBS4 | 1 | ON-OFF |  |
| Electron transfer flavoprotein subunit alpha, mitochondrial OS=Homo sapiens GN=ETFA PE=1 SV=1 | P13804 | 1 | 10 |  |
| Elongation factor 1-beta OS=Homo sapiens GN=EEF1B2 PE=1 SV=3 | P24534 | 1 | ON-OFF | 10 |
| Endothelial differentiation-related factor 1 OS=Homo sapiens GN=EDF1 PE=1 SV=1 | O60869 | 1 |  | 10 |
| ERO1-like protein alpha OS=Homo sapiens GN=ERO1L PE=1 SV=2 | Q96HE7 | 1 | ON |  |
| Eukaryotic initiation factor 4A-II OS=Bos taurus GN=EIF4A2 PE=2 SV=1 | Q3SZ65 | 1 | ON |  |
| Eukaryotic translation initiation factor 1b OS=Macaca fascicularis GN=EIF1B PE=3 SV=1 | Q4R4X9 | 1 | ON-10 |  |
| Eukaryotic translation initiation factor 4 gamma 1 OS=Mus musculus GN=Eif4g1 PE=1 SV=1 | Q6NZJ6 | 1 | 10 |  |
| F-actin-capping protein subunit alpha-1 OS=Macaca fascicularis GN=CAPZA1 PE=2 SV=1 | Q4R959 | 1 | ON-10 |  |
| Ferritin heavy chain OS=Sus scrofa GN=FTH1 PE=1 SV=3 | P19130 | 1 | 10 |  |
| Ferritin light chain OS=Homo sapiens GN=FTL PE=1 SV=2 | P02792 | 1 | OFF |  |
| FIC domain-containing protein OS=Mus musculus GN=Ficd PE=2 SV=1 | Q8BIX9 | 1 | ON |  |
| Fructose-bisphosphate aldolase A OS=Rattus norvegicus GN=Aldoa PE=1 SV=2 | P05065 | 1 | ON-OFF-10 |  |
| Galectin-related protein OS=Homo sapiens GN=GRP PE=1 SV=2 | Q3ZCW2 | 1 | ON |  |
| Gap junction alpha-1 protein OS=Canis familiaris GN=GJA1 PE=2 SV=3 | Q6S9C0 | 1 |  | OFF |
| Glucosylceramidase OS=Sus scrofa GN=GBA PE=3 SV=1 | Q70KH2 | 1 | OFF |  |
| Glutamate dehydrogenase 1, mitochondrial (Fragments) OS=Sus scrofa GN=GLUD1 PE=1 SV=1 | P42174 | 1 |  | 10 |
| Golgi reassembly-stacking protein 2 OS=Mus musculus GN=Gorasp2 PE=1 SV=3 | Q99JX3 | 1 | 10 |  |
| Golgin subfamily A member 3 OS=Homo sapiens GN=GOLGA3 PE=1 SV=2 | Q08378 | 1 | ON |  |
| H/ACA ribonucleoprotein complex subunit 2 OS=Bos taurus GN=NOLA2 PE=2 SV=1 | Q5E950 | 1 | ON-OFF |  |
| H/ACA ribonucleoprotein complex subunit 3 OS=Mus musculus GN=Nola3 PE=2 SV=1 | Q9CQS2 | 1 | ON-OFF |  |
| Heat shock 70 kDa protein 1B OS=Bos mutus grunniens GN=HSPA1B PE=2 SV=1 | Q4U0F3 | 1 | 10 |  |
| Heat shock protein HSP 90-beta OS=Equus caballus GN=HSP90AB1 PE=2 SV=3 | Q9GKX8 | 1 | ON |  |
| Heme oxygenase 1 OS=Sus scrofa GN=HMOX1 PE=2 SV=1 | P32394 | 1 | ON-OFF |  |
| Hemoglobin subunit alpha OS=Mus musculus GN=Hba PE=1 SV=2 | P01942 | 1 | ON |  |
| Hemoglobin subunit alpha-2 OS=Bos mutus grunniens PE=1 SV=1 | P01968 | 1 | ON |  |
| Heterochromatin protein 1-binding protein 3 OS=Bos taurus GN=HP1BP3 PE=2 SV=1 | Q08DU9 | 1 | 10 |  |
| High mobility group protein HMGI-C OS=Homo sapiens GN=HMGA2 PE=1 SV=1 | P52926 | 1 |  | 10 |
| Histone deacetylase complex subunit SAP18 OS=Homo sapiens GN=SAP18 PE=1 SV=1 | O00422 | 1 | OFF |  |
| Histone H1.0 OS=Homo sapiens GN=H1F0 PE=1 SV=3 | P07305 | 1 |  | OFF-10 |
| Histone H1.1 OS=Homo sapiens GN=HIST1H1A PE=1 SV=3 | Q02539 | 1 |  | OFF-10 |
| Huntingtin-interacting protein K OS=Macaca fascicularis GN=HYPK PE=2 SV=1 | Q2PFU1 | 1 |  | 10 |
| Immunity-related GTPase family M protein OS=Rattus norvegicus GN=Irgm PE=2 SV=1 | Q6AYC2 | 1 |  | OFF |
| Importin subunit alpha-6 OS=Homo sapiens GN=KPNA5 PE=1 SV=1 | O15131 | 1 |  | OFF |
| Interferon regulatory factor 3 OS=Sus scrofa GN=IRF3 PE=2 SV=1 | Q764M6 | 1 | OFF-10 |  |
| Junctophilin-2 OS=Mus musculus GN=Jph2 PE=1 SV=2 | Q9ET78 | 1 |  | 10 |
| KH domain-containing, RNA-binding, signal transduction-associated protein 1 OS=Homo sapiens GN=KHDRBS1 PE=1 SV=1 | Q07666 | 1 | OFF |  |
| L-lactate dehydrogenase A-like 6B OS=Bos taurus GN=LDHAL6B PE=2 SV=1 | Q3T056 | 1 | ON |  |
| Lamina-associated polypeptide 2 isoforms alpha/zeta OS=Mus musculus GN=Tmpo PE=1 SV=3 | Q61033 | 1 | ON | 10 |
| Laminin subunit beta-1 OS=Homo sapiens GN=LAMB1 PE=1 SV=1 | P07942 | 1 |  | 10 |
| Leucine zipper protein 1 OS=Mus musculus GN=Luzp1 PE=1 SV=2 | Q8R4U7 | 1 | ON |  |
| LIM domain and actin-binding protein 1 OS=Homo sapiens GN=LIMA1 PE=1 SV=1 | Q9UHB6 | 1 | ON |  |
| Macrophage mannose receptor 2 OS=Homo sapiens GN=MRC2 PE=1 SV=1 | Q9UBG0 | 1 | OFF |  |
| Methyl-CpG-binding protein 2 OS=Rattus norvegicus GN=Mecp2 PE=1 SV=1 | Q00566 | 1 |  | OFF-10 |
| Mitochondrial fission 1 protein OS=Homo sapiens GN=FIS1 PE=1 SV=2 | Q9Y3D6 | 1 | 10 |  |
| Mitochondrial import inner membrane translocase subunit Tim10 OS=Homo sapiens GN=TIMM10 PE=1 SV=1 | P62072 | 1 | ON-OFF-10 |  |
| Mitochondrial import inner membrane translocase subunit Tim13 OS=Mus musculus GN=Timm13 PE=1 SV=1 | P62075 | 1 | OFF-10 |  |
| Mitochondrial import inner membrane translocase subunit Tim9 OS=Homo sapiens GN=TIMM9 PE=1 SV=1 | Q9Y5J7 | 1 | 10 |  |
| Mitochondrial import receptor subunit TOM70 OS=Homo sapiens GN=TOMM70A PE=1 SV=1 | O94826 | 1 | ON |  |
| Myotrophin OS=Canis familiaris GN=MTPN PE=3 SV=3 | Q863Z4 | 1 | OFF-10 |  |
| N-acetylglucosamine-6-sulfatase OS=Capra hircus GN=GNS PE=2 SV=1 | P50426 | 1 | 10 |  |
| NAC-alpha domain-containing protein 1 OS=Homo sapiens GN=NACAD PE=2 SV=2 | O15069 | 1 | ON-OFF-10 |  |
| Neurabin-1 OS=Homo sapiens GN=PPP1R9A PE=1 SV=2 | Q9ULJ8 | 1 |  | OFF-10 |
| Non-histone chromosomal protein HMG-17 OS=Canis familiaris GN=HMGN2 PE=3 SV=3 | Q711A6 | 1 |  | 10 |
| NSFL1 cofactor p47 OS=Homo sapiens GN=NSFL1C PE=1 SV=2 | Q9UNZ2 | 1 | ON-10 |  |
| Nuclear pore complex protein Nup93 OS=Mus musculus GN=Nup93 PE=2 SV=1 | Q8BJ71 | 1 | 10 |  |
| Nuclear receptor-interacting protein 1 OS=Mus musculus GN=Nrip1 PE=1 SV=1 | Q8CBD1 | 1 | ON-10 |  |
| Nucleolar phosphoprotein p130 OS=Homo sapiens GN=NOLC1 PE=1 SV=2 | Q14978 | 1 | ON |  |
| Nucleolar protein 5A OS=Macaca fascicularis GN=NOL5A PE=2 SV=1 | Q95K50 | 1 | 10 |  |
| Nucleoside diphosphate kinase 3 OS=Mus musculus GN=Nme3 PE=2 SV=3 | Q9WV85 | 1 | ON |  |
| Obg-like ATPase 1 OS=Mus musculus GN=Ola1 PE=1 SV=1 | Q9CZ30 | 1 | ON |  |
| Oligoribonuclease, mitochondrial OS=Bos taurus GN=REXO2 PE=2 SV=1 | A2VE52 | 1 | ON |  |
| Paraspeckle component 1 OS=Homo sapiens GN=PSPC1 PE=1 SV=1 | Q8WXF1 | 1 | ON |  |
| Parathymosin OS=Homo sapiens GN=PTMS PE=2 SV=2 | P20962 | 1 | 10 |  |
| PDZ and LIM domain protein 1 OS=Rattus norvegicus GN=Pdlim1 PE=2 SV=4 | P52944 | 1 | ON |  |
| PDZ and LIM domain protein 2 OS=Bos taurus GN=PDLIM2 PE=2 SV=1 | Q3T0C8 | 1 | OFF-10 |  |
| PDZ and LIM domain protein 7 OS=Homo sapiens GN=PDLIM7 PE=1 SV=1 | Q9NR12 | 1 | ON |  |
| Phosducin-like protein 3 OS=Mus musculus GN=Pdcl3 PE=1 SV=1 | Q8BVF2 | 1 |  | 10 |
| Phosphoribosyltransferase domain-containing protein 1 OS=Homo sapiens GN=PRTFDC1 PE=1 SV=1 | Q9NRG1 | 1 | ON |  |
| Poly(rC)-binding protein 1 OS=Homo sapiens GN=PCBP1 PE=1 SV=2 | Q15365 | 1 | OFF |  |
| Poly(rC)-binding protein 1 OS=Homo sapiens GN=PCBP1 PE=1 SV=2 | Q15365 | 1 | 10 |  |
| Poly(U)-binding-splicing factor PUF60 OS=Homo sapiens GN=PUF60 PE=1 SV=1 | Q9UHX1 | 1 | ON-OFF |  |
| Pre-mRNA-processing factor 19 OS=Homo sapiens GN=PRPF19 PE=1 SV=1 | Q9UMS4 | 1 | OFF |  |
| Prefoldin subunit 5 OS=Homo sapiens GN=PFDN5 PE=1 SV=2 | Q99471 | 1 | 10 |  |
| Prohibitin OS=Homo sapiens GN=PHB PE=1 SV=1 | P35232 | 1 | OFF-10 |  |
| Proteasome assembly chaperone 1 OS=Homo sapiens GN=PSMG1 PE=1 SV=1 | O95456 | 1 | ON-OFF |  |
| Proteasome subunit alpha type-2 OS=Homo sapiens GN=PSMA2 PE=1 SV=2 | P25787 | 1 | OFF |  |
| Proteasome subunit alpha type-6 OS=Bos taurus GN=PSMA6 PE=1 SV=1 | Q2YDE4 | 1 | ON-OFF |  |
| Protein disulfide-isomerase A4 OS=Homo sapiens GN=PDIA4 PE=1 SV=2 | P13667 | 1 | ON-OFF |  |
| Protein enabled homolog OS=Mus musculus GN=Enah PE=1 SV=2 | Q03173 | 1 | 10 |  |
| Protein FAM103A1 OS=Pongo abelii GN=FAM103A1 PE=3 SV=1 | Q5R9Q6 | 1 |  | OFF |
| Protein FAM54B OS=Pongo abelii GN=FAM54B PE=2 SV=1 | Q5R3Z9 | 1 | 10 |  |
| Protein PRR15 OS=Mus musculus GN=Prr15 PE=2 SV=1 | Q9D1T5 | 1 |  | 10 |
| Protein S100-A10 OS=Homo sapiens GN=S100A10 PE=1 SV=2 | P60903 | 1 | OFF-10 |  |
| Protein S100-A11 OS=Sus scrofa GN=S100A11 PE=1 SV=1 | P31950 | 1 | ON-10 |  |
| Protein S100-A4 OS=Canis familiaris GN=S100A4 PE=3 SV=1 | Q9TV56 | 1 | ON-OFF-10 |  |
| Protein S100-A6 OS=Mus musculus GN=S100a6 PE=1 SV=3 | P14069 | 1 | ON-OFF-10 | ON-OFF |
| Protein transport protein Sec23B OS=Mus musculus GN=Sec23b PE=2 SV=1 | Q9D662 | 1 | OFF |  |
| Protein transport protein Sec61 subunit gamma OS=Canis familiaris GN=SEC61G PE=1 SV=1 | P60058 | 1 | OFF-10 |  |
| Protocadherin beta-4 OS=Pan troglodytes GN=PCDHB4 PE=3 SV=1 | Q5DRD0 | 1 |  | 10 |
| Purkinje cell protein 4-like protein 1 OS=Mus musculus GN=Pcp4l1 PE=2 SV=1 | Q6W8Q3 | 1 | OFF |  |
| Putative histone H3-like OS=Mus musculus PE=5 SV=2 | P02301 | 1 | ON |  |
| Putative RNA-binding protein Luc7-like 1 OS=Mus musculus GN=Luc7l PE=2 SV=2 | Q9CYI4 | 1 |  | 10 |
| Putative RNA-binding protein Luc7-like 2 OS=Mus musculus GN=Luc7l2 PE=1 SV=1 | Q7TNC4 | 1 |  | 10 |
| Pyruvate dehydrogenase protein X component, mitochondrial OS=Homo sapiens GN=PDHX PE=1 SV=3 | O00330 | 1 | OFF-10 |  |
| Ras GTPase-activating protein-binding protein 2 OS=Pongo abelii GN=G3BP2 PE=2 SV=1 | Q5R9L3 | 1 | OFF-10 |  |
| Ras-related protein Rap-1A OS=Homo sapiens GN=RAP1A PE=1 SV=1 | P62834 | 1 | ON-OFF-10 |  |
| Reticulocalbin-2 OS=Homo sapiens GN=RCN2 PE=1 SV=1 | Q14257 | 1 |  | 10 |
| Ribulose-phosphate 3-epimerase OS=Mus musculus GN=Rpe PE=2 SV=1 | Q8VEE0 | 1 | ON |  |
| RNA-binding protein 39 OS=Mus musculus GN=Rbm39 PE=1 SV=1 | Q8VH51 | 1 |  |  |
| RNA-binding protein FUS OS=Homo sapiens GN=FUS PE=1 SV=1 | P35637 | 1 | OFF |  |
| Scaffold attachment factor B1 OS=Pongo abelii GN=SAFB PE=2 SV=1 | Q5R452 | 1 |  | OFF-10 |
| Selenide, water dikinase 1 OS=Homo sapiens GN=SEPHS1 PE=1 SV=2 | P49903 | 1 | OFF |  |
| Selenoprotein M OS=Homo sapiens GN=SELM PE=2 SV=3 | Q8WWX9 | 1 | ON-OFF-10 |  |
| Serine protease HTRA1 OS=Homo sapiens GN=HTRA1 PE=1 SV=1 | Q92743 | 1 | OFF |  |
| Serine/threonine-protein phosphatase 2A 56 kDa regulatory subunit beta isoform OS=Oryctolagus cuniculus GN=PPP2R5B PE=2 SV=1 | Q28647 | 1 |  | 10 |
| Seryl-tRNA synthetase, cytoplasmic OS=Homo sapiens GN=SARS PE=1 SV=3 | P49591 | 1 |  | 10 |
| SH3 domain-binding glutamic acid-rich-like protein 3 OS=Homo sapiens GN=SH3BGRL3 PE=1 SV=1 | Q9H299 | 1 | ON |  |
| SHC-transforming protein 1 OS=Homo sapiens GN=SHC1 PE=1 SV=4 | P29353 | 1 | 10 |  |
| Small nuclear ribonucleoprotein E OS=Bos taurus GN=SNRPE PE=3 SV=1 | A4FUI2 | 1 | ON-OFF |  |
| Small ubiquitin-related modifier 2 OS=Cricetulus griseus GN=SUMO2 PE=3 SV=1 | Q6LDZ8 | 1 | ON-OFF-10 |  |
| Sodium/potassium-transporting ATPase subunit beta-3 OS=Homo sapiens GN=ATP1B3 PE=1 SV=1 | P54709 | 1 | OFF-10 |  |
| Spliceosome RNA helicase BAT1 OS=Canis familiaris GN=BAT1 PE=3 SV=1 | Q5WR10 | 1 | ON |  |
| Splicing factor 3 subunit 1 OS=Homo sapiens GN=SF3A1 PE=1 SV=1 | Q15459 | 1 |  | 10 |
| Splicing factor U2AF 35 kDa subunit OS=Homo sapiens GN=U2AF1 PE=1 SV=3 | Q01081 | 1 | ON-OFF |  |
| Succinate dehydrogenase [ubiquinone] iron-sulfur subunit, mitochondrial OS=Bos taurus GN=SDHB PE=2 SV=1 | Q3T189 | 1 | ON |  |
| Superoxide dismutase [Cu-Zn] OS=Sus scrofa GN=SOD1 PE=1 SV=2 | P04178 | 1 | 10 |  |
| Surfeit locus protein 4 OS=Homo sapiens GN=SURF4 PE=2 SV=3 | O15260 | 1 | OFF-10 |  |
| Synaptic vesicle membrane protein VAT-1 homolog OS=Mus musculus GN=Vat1 PE=2 SV=3 | Q62465 | 1 | ON |  |
| T-complex protein 1 subunit gamma OS=Bos taurus GN=CCT3 PE=2 SV=1 | Q3T0K2 | 1 | ON |  |
| THAP domain-containing protein 5 OS=Bos taurus GN=THAP5 PE=2 SV=2 | Q1RMM0 | 1 | OFF | 10 |
| Thioredoxin domain-containing protein 12 OS=Homo sapiens GN=TXNDC12 PE=1 SV=1 | O95881 | 1 | ON-10 |  |
| Thioredoxin domain-containing protein 17 OS=Pongo abelii GN=TXNDC17 PE=2 SV=1 | Q5REA8 | 1 | ON |  |
| Thioredoxin-dependent peroxide reductase, mitochondrial OS=Bos taurus GN=PRDX3 PE=1 SV=2 | P35705 | 1 | ON-OFF-10 |  |
| Threonyl-tRNA synthetase, cytoplasmic OS=Bos taurus GN=TARS PE=2 SV=1 | Q3ZBV8 | 1 |  | 10 |
| THUMP domain-containing protein 2 OS=Homo sapiens GN=THUMPD2 PE=2 SV=1 | Q9BTF0 | 1 | 10 |  |
| Thymosin beta-4 OS=Bos taurus GN=TMSB4 PE=1 SV=2 | P62326 | 1 | ON-OFF-10 |  |
| Transcription intermediary factor 1-beta OS=Mus musculus GN=Trim28 PE=1 SV=3 | Q62318 | 1 | 10 |  |
| Translocon-associated protein subunit alpha OS=Canis familiaris GN=SSR1 PE=1 SV=1 | P16967 | 1 | ON | 10 |
| Transthyretin OS=Bos taurus GN=TTR PE=2 SV=1 | O46375 | 1 | ON |  |
| Tubulin folding cofactor B OS=Homo sapiens GN=TBCB PE=1 SV=2 | Q99426 | 1 | 10 |  |
| Type II inositol-3,4-bisphosphate 4-phosphatase OS=Mus musculus GN=Inpp4b PE=2 SV=1 | Q6P1Y8 | 1 | 10 |  |
| U4/U6.U5 tri-snRNP-associated protein 1 OS=Rattus norvegicus GN=Sart1 PE=2 SV=1 | Q5XIW8 | 1 | 10 |  |
| Ubiquinone biosynthesis protein COQ4 homolog OS=Rattus norvegicus GN=Coq4 PE=2 SV=1 | Q4FZU1 | 1 | ON |  |
| Uncharacterized protein C6orf115 OS=Homo sapiens GN=C6orf115 PE=1 SV=1 | Q9P1F3 | 1 | OFF |  |
| Uncharacterized protein KIAA0825 homolog OS=Mus musculus PE=2 SV=2 | Q3UPC7 | 1 |  | 10 |
| Vesicle-associated membrane protein-associated protein B OS=Sus scrofa GN=VAPB PE=3 SV=1 | A5GFS8 | 1 | 10 |  |
| Vesicle-trafficking protein SEC22b OS=Homo sapiens GN=SEC22B PE=1 SV=3 | O75396 | 1 | OFF-10 |  |
| Zinc finger protein 207 OS=Pongo abelii GN=ZNF207 PE=2 SV=1 | Q5R8K4 | 1 |  | ON-OFF-10 |
| Zinc finger ZZ-type and EF-hand domain-containing protein 1 OS=Mus musculus GN=Zzef1 PE=1 SV=2 | Q5SSH7 | 1 | OFF |  |

a) Accession numbers for SwissProt database (Mammalia); b) number of matched peptides; c) samples (ON, OFF, 10 min) in which each protein has been identified after anion-exchange chromatography; d) samples (ON, OFF, 10 min) in which each protein has been identified after cation-exchange chromatography.

Only proteins identified in all technical replicates are reported.
